# Fecal microbiota transplantation in irritable bowel syndrome: A meta-analysis of randomized controlled trials

**DOI:** 10.3389/fmed.2022.1039284

**Published:** 2022-11-03

**Authors:** Chatpol Samuthpongtorn, Piyawat Kantagowit, Rapat Pittayanon, Tanisa Patcharatrakul, Sutep Gonlachanvit

**Affiliations:** ^1^Faculty of Medicine, Chulalongkorn University, Bangkok, Thailand; ^2^Division of Gastroenterology, Department of Medicine, Faculty of Medicine, Chulalongkorn University and King Chulalongkorn Memorial Hospital, The Thai Red Cross Society, Bangkok, Thailand; ^3^Center of Excellence in Neurogastroenterology and Motility, Department of Medicine, Faculty of Medicine, Chulalongkorn University, Bangkok, Thailand

**Keywords:** dysbiosis, irritable bowel syndrome, fecal microbiota transplantation, gastrointestinal diseases, meta-analysis

## Abstract

**Introduction:**

Fecal microbiota transplantation (FMT) has been proposed as a potential treatment for irritable bowel syndrome (IBS); however, the consensus regarding its efficacy and safety is limited.

**Materials and Methods:**

We performed a systematic search of the literature using PubMed, EMBASE, Ovid MEDLINE, and Cochrane. Meta-analyses were conducted in relative risk (RR) or standard mean difference (SMD) using 95% confidence intervals (CI). Cochrane risk-of-bias 2 tool (RoB2) was employed to evaluate the study quality.

**Result:**

Of 2,589 potential records, 7 studies with 9 cohorts involving 505 participants were included. Meta-analyses showed no significant difference in the short-term (12 weeks) and long-term (12 months) global improvement of IBS symptoms of FMT vs. placebo (RR 0.63, 95% CI 0.39–1.00 and RR 0.88, 95% CI 0.53–1.45, respectively). There were statistically significant differences of short-term IBS-SSS improvement (SMD –0.58, 95% CI –1.09 to –0.88) and short-term IBS-QoL improvement (SMD 0.67, 95% CI 0.43–0.91). Eight from 9 studies (88.9%) had a low risk of bias. The subgroup analysis revealed the short-term global symptoms improvement in studies with low-risk of bias (RR 0.53, 95% CI 0.35–0.81), studies with well-defined donors (RR 0.31, 95% CI 0.14–0.72), and studies with FMT using colonoscopy (RR 0.66, 95% CI 0.47–0.92). Major FMT adverse events are transient and rapidly self-limiting.

**Conclusion:**

FMT significantly improved IBS-SSS and IBS-QoL in the short-term period in IBS patients. However, global symptom improvement showed no significance. Well-defined donors and appropriate fecal administration routes appear to be important factors for the successful outcomes of FMT in IBS.

**Systematic review registration:**

[www.crd.york.ac.uk/prospero], identifier [CRD42021246101].

## Introduction

Irritable bowel syndrome (IBS) is a clinical-based functional bowel disorder characterized by abdominal pain and altered bowel habits in the absence of structural abnormalities (1, 2). The pooled regional prevalence of IBS ranges from 5.8 to 17.5% worldwide (3). The disorder significantly impairs health-related quality of life (QoL), reduces work productivity, and results in high healthcare costs (4–6). The pathogenesis of IBS is heterogeneous, contributing to critical challenges in advancing successful therapeutic strategies (7).

Recent studies have highlighted an important role of the gut microbiota in patients with IBS, which diminishes in microbial biodiversity compared with healthy controls (8–13). Manipulation of the gut microbiota has been suggested as a therapeutic approach to managing IBS symptoms and reinforced by increasing data from clinical studies using prebiotics, probiotics, synbiotics, antibiotics, and dietary modifications (14–16).

Recent randomized studies of fecal microbiota transplantation (FMT) in IBS have shown significant results on relieving symptoms compared to placebo (17, 18). Despite that, prior RCTs have variations in FMT methods which may lead to heterogenous results of the trials, so that results from these RCTs have been inconsistent (18–21). The important factors including route of administration, characteristics of stool donors, donor microbiome profile, and patient microbiome profile influence the efficacy of FMT in IBS (17, 18, 22). Recent meta-analyses of RCTs on the efficacy of FMT in IBS have been published (23, 24). These meta-analyses demonstrated that FMT does not result in a significant global improvement in IBS patients, but that FMT performed *via* invasive routes significantly improved global IBS symptoms. However, neither the characteristics of stool donors nor the significance of stool donor inclusion were discussed in detail. Moreover, although the microbiome profile was mentioned, the association between the microbiome profile and global symptoms were unclear. Therefore, this study aimed to conduct a systematic review and meta-analyses of RCTs to estimate the efficacy and safety of FMT for the treatment of IBS, with subgroup analyses by route of FMT administration, type of feces used in FMT, donors’ microbiome profiles, patients’ microbiome profiles, and quality of stool donors.

## Materials and methods

We conducted a systematic review and meta-analysis following the recommendations of the Preferred Reporting Items for Systematic Review and Meta-Analysis Protocols (PRISMA-P) 2015 statement (25). We prospectively registered the systematic review with PROSPERO International Prospective Register of Ongoing Systematic Reviews (Registration number: CRD42021246101).

### Search strategy

We performed a systematic search of the literature using PubMed, EMBASE, Ovid MEDLINE, and Cochrane. The term of the search strategy is presented in detail in Supplementary Appendix 1.

### Study selection and patient population

The inclusion criteria were randomized-controlled trials of patients with IBS defined by Rome I, Rome II, Rome III, or Rome IV compared FMT with placebo. Both adult and pediatric studies were allowed. The exclusion criteria were: (1) studies failing to report our outcomes of interest (2) type of study designs which are review article, protocol, letter, comment, guidelines, case-control, or cohort studies. (3) Not yet published as full manuscript or not peer-reviewed, and (4) not in the English language.

### Outcomes of interest

The primary outcome was the global improvement in IBS symptoms after FMT. Global improvement was defined as dichotomous assessment in the form of either an assessment of global symptom cure or improvement, or abdominal pain cure or improvement, after completion of therapy. It was also defined as a dichotomous response determined from the IBS Severity Scoring System instrument (IBS-SSS) score or Gastrointestinal Symptom Rating Scale for IBS (GSRS-IBS) total score using a predefined cut point of response and non-response. The secondary outcomes were the improvement of IBS-SSS (reduction in IBS-SSS score), the improvement of QoL (score increase on IBS-QoL), and adverse events (AEs). Subgroup analyses of the primary outcome were conducted by study characteristics including the risk of bias, characteristic of donors (well-defined, relatively well-defined, or unclearly defined donors), route of FMT administration (capsules, nasogastric tube, gastroscopy, or colonoscopy), type of feces used in FMT (fresh or frozen), and microbiome profiles. We gathered data for two time periods: the short term and the long term. Eight to 12 weeks’ outcomes were classified as “short-term” and 6–12 months as “long-term” (26, 27). We collected data from each research to determine the longest durations for both short- and long-term periods. For instance, if research reports findings for both 8 and 12 weeks, we would choose the 12-week outcome for meta-analyses.

### Eligibility assessment

Two independent reviewers (C.S., P.K.) screened articles for eligible studies and then extracted data from eligible published articles of FMT in patients with IBS. Discrepancies between two reviewers were re-checked and discussed to reach a consensus. If the authors were unable to reach a consensus, the third-person GI specialist (R.P.) reached the judgment.

The data collection included: (1) study characteristics including authors, study type, country, (2) patients characteristics including mean age, sex, IBS criteria, IBS subtype, the number of patients, IBS severity, the year with IBS, current medication, and naïve or refractory to standard treatment, (3) stool donor including the number of donors, inclusion and exclusion criteria, stool preparation, (4) placebo preparation, (5) primary and secondary outcomes, (6) details of FMT methods, including preparation for FMT, the FMT route, the frequency and duration of FMT, (7) the duration of follow-up after FMT, and (8) FMT-related adverse events. We did contact the corresponding authors to request incompletely reported data on the outcomes of interest. If we could not get the response by 14 days, the analyses would be conducted by using available data.

### Assessment of quality of evidence

We applied the Cochrane risk-of-bias 2 (RoB2) tool for evaluating the quality of each eligible study in terms of randomization process, allocation concealment, blinding of participants, personnel and outcome assessment, complete outcome data addressed, selective outcome reporting, and other sources of bias.

### Statistical analysis

The pooled effect sizes and 95% confidence intervals (CI) were calculated using random-effects models. Meta-analyses were conducted using the relative risk (RR) method for dichotomous outcomes and the standard mean difference (SMD) method for continuous outcomes. *P*-value < 0.05 was considered statistically significant. Heterogeneity was determined using the Cochran’s *Q*-test [a *p*-value of 0.10 indicated heterogeneity] and the Higgins’ test [*I*^2^] [low heterogeneity was defined as less than 25%, moderate heterogeneity was defined as 25–75%, and high heterogeneity was defined as more than 75%] (28). If a published study reported more than one method of intervention (e.g., different amount of FMT) or cross-over trial, the data from that study was extracted into two separate “cohorts.” Thus, our meta-analysis used the terms “study” and “cohort” to represent these definitions. Subgroup analyses were conducted by study characteristics such as the risk of bias, route of FMT administration (capsules, nasogastric tube, gastroscopy, or colonoscopy), type of feces used in FMT (fresh or frozen), microbiome profiles, and quality of donors (well, relatively well, and unclearly defined donors). Additionally, sensitivity analyses were considered repeating the meta-analysis to determine the statistical robustness of the primary outcome by removing one study at a time. As the number of identified studies was fewer than 10, the Egger’s regression asymmetry test and funnel plots were considered with caution to evaluate publication bias using STATA 16.0 (StataCorp, TX, USA). RevMan 5.4 (The Cochrane Collaboration, The Nordic Cochrane Centre, Copenhagen, Denmark) was used to conduct the meta-analysis.

### Microbiome analysis

Microbiome subgroup analyses of the primary outcome were planned to be performed by microbiome profiles, including recipients’ baseline microbiota, donors’ baseline microbiota, and the difference of baseline microbiota between donors and recipients. In addition, the association of the change in recipients’ microbiome profiles after FMT, the difference of dysbiosis index of recipients after FMT, as well as other gut microbiome profiles (e.g., specific species of bacteria) of recipients after FMT and the primary outcome would be scrutinized using meta-analysis.

### Stool donor classification

Stool donor subgroup analyses of the primary outcome were planned to be performed by classifying the donors into “well-defined donors,” “relatively well-defined donors,” and “unclearly defined donors” using the following 3 main factors. In the first factor, we utilized the standard donor selection and collection criteria for FMT in clinical practice to reduce the risk of disease transmission (29–32) to ensure the safety of the stool derived from the donors. In the second factor, we concentrated on the characteristics of the donor microbiome profiles that likely to be suitable for IBS patients including high microbiome diversity and high amount of butyrate-producing bacteria, and in the third factor, we concentrated on the factors that ensure the stability of the donor microbiota profiles that had been collected during the study.

**(1) The donor selection and collection process.**

The stool donors were assessed for general health status and gastrointestinal conditions by clinical assessment, and serological and fecal tests to minimize the risk of infection or other disease transmission. The donor selection had to meet at least one of the following protocol/guidelines**; (a) The protocol** defined by Kim and Gluck (29), Bakken et al. (30), Brandt (31), and McCune et al. (32) (Supplementary Table 1), **(b) one of the following international guidelines**: European consensus conference on FMT in clinical practice (33), A joint British Society of Gastroenterology and Healthcare Infection Society guidelines (34), International consensus conference on stool banking for FMT in clinical practice (35), A joint document of Asia-Pacific Association of Gastroenterology and Asia-Pacific Society for Digestive Endoscopy (36), Australian consensus statements for the regulation, production, and use of FMT in clinical practice (37).

**(2) The donor microbiome profile.**

The donor microbiome profile had to be determined *via* direct and indirect assessment. **The direct assessment** was determined by at least one of the following conditions; (a) high microbiome diversity or microbiome richness (17, 18) and (b) high amount of butyrate-producing bacteria (38) **Whereas the indirect assessment was determined by that the studies control the factors** that influence donor microbiome diversity, such as breastfeeding, bowel movement, vaginal delivery, and antibiotic-free status (17, 22).

**(3) The stability of the donor microbiome composition.**

The stability of the microbiome composition was determined *via* direct and indirect assessment. **The direct assessment** was determined by the stability of microbiome composition throughout the duration of the study measured by gut microbiome diversity (17, 18). **The indirect assessment** was determined by the studies which control the donor health and behaviors to be standardized throughout the study (e.g., prohibited from taking medication, prohibited to travel abroad, etc. (18, 22).

If the donors met all 3 criteria with sufficient information for direct assessment, they were classified as the “well-defined donor.” If the donors seemed to meet all 3 criteria but had insufficient information for direct assessment, they were sorted as the “relatively well-defined donor.” On condition that the description of the donor recruitment process was inadequate to decide whether the donors fell into one of the other categories, they would be categorized as the “unclearly defined donor.” The criteria of well-defined donor were illustrated in Table 1.

**TABLE 1:** Criteria of well-defined donor.

Criteria of well-defined donor
**1. Donor selection and collection process met** at least one of the following protocol/guidelines −**The protocol** defined by Kim and Gluck (29), Bakken et al. (30), Brandt (31), and Mccune et al. (32) (Supplementary Table 1) −**One of the following international guidelines:** ° European consensus conference on fecal microbiota transplantation in clinical practice (33) ° Joint British Society of Gastroenterology and Healthcare Infection Society guidelines (34) ° International consensus conference on stool banking for fecal microbiota transplantation in clinical practice (35) ° Joint document of Asia-Pacific Association of Gastroenterology and Asia-Pacific Society for Digestive Endoscopy (36) ° Australian consensus statements for the regulation, production, and use of fecal microbiota transplantation in clinical practice (37)
**2. Donor microbiome profile** **a. Direct assessment** was determined by at least one of the following conditions - **The high microbiome diversity or microbiome richness of donors** (the microbiome diversity or microbiome richness of donor suspensions used for FMT being statistically significantly greater than that of patients with irritable bowel syndrome (*p* < 0.05) - **The high amount of butyrate-producing bacteria of donor** (the relative abundance of the butyrate-producing bacteria in the baseline samples of the donors was higher than in the fecal samples of the patients (*p* < 0.05)) And **b. Indirect assessment** was determined by that **the studies control the factors that influence donor microbiome diversity**, such as breastfeeding, bowel movement, vaginal delivery, and antibiotic-free status
**3. Donor stability of the microbiome composition** **a. Direct assessment** was determined by **the stability of microbiome composition throughout the duration of the study measured by gut microbiome diversity** (compositions of the different samples donated by donors over time were remarkably similar when evaluated by using diversity measures such as Euclidean distance, Unifrac distance, etc.) And **b. Indirect assessment** was determined by that **the studies which control the donor health and behaviors to be standardized throughout the duration of the study** (e.g., prohibited to take medication, prohibited to travel abroad, etc.

Donor recruitment process met all 3 criteria with direct assessment: **Well-defined donor.** Donor recruitment process met all 3 criteria but have insufficient information for direct assessment: **Relatively well-defined donor.** Donor recruitment process was inadequate to decide whether the donors fell into one of the other categories: **Unclearly defined donor.**

## Results

**FIGURE 1:**
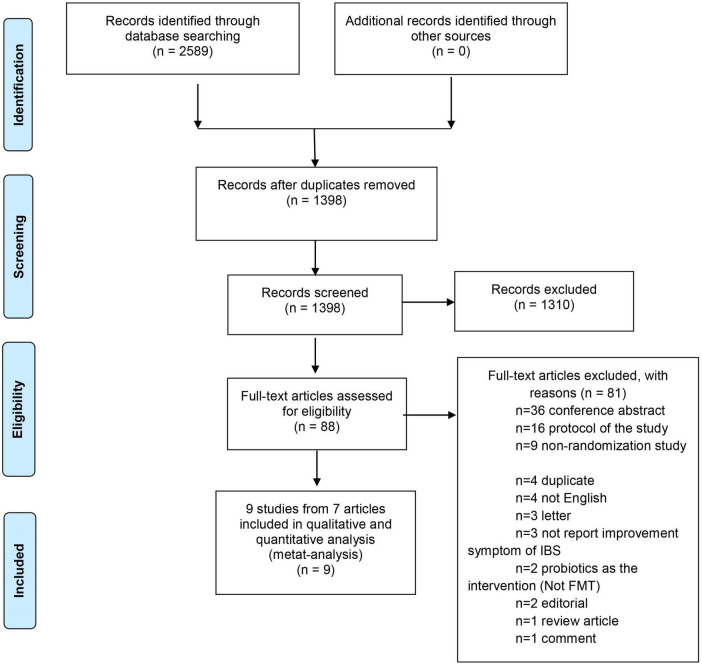
PRISMA diagram.

The database search identified 2,589 potential records. After removing duplicates, 1,398 titles were left for the screening phase. Of these, 88 theme-related studies passed the initial screen and were further assessed for eligibility with full-text articles. Eighty-one studies were excluded as the following: 36 conference abstracts, 16 study protocols, 9 non-randomization studies, 4 duplicate, 4 not English, 3 letters, 3 not report improvement of IBS symptoms, 2 used probiotics as the intervention (Not FMT), 2 editorials, 1 review article, and 1 comment article. Therefore, 7 articles with 9 studies were eligible for the data synthesis (17–21, 38, 39) (Figure 1). Summary of the baseline characteristics and outcomes of the included studies was shown in Table 2. Summary of other descriptive characteristics of the selected studies was shown in Supplementary Table 2.

**TABLE 2:** Summary of the descriptive characteristics of the included studies.

Author, year	Country	Number of center	IBS patients									Primary outcomes	Secondary outcomes
			IBS subtype	IBS criteria	Mean age (S.D.)		Male gender, *n* (%)			IBS-SSS baseline (mean with SD or median with IQR)			
					FMT	Placebo	Total	FMT	Placebo	FMT	Placebo		
(21, 21)	USA	3	IBS-D	ROME III	36.2 (16.5) ^†^	39.1 (15.8) ^†^	30 (62.5%)	16 (64%)	14 (61%)	282 (65)	309 (64)	Decrease in IBS-SSS ≥ 50 points at 3 months	Change in IBS-QoL, HADS, Bristol stool scale scores, microbiome profile, and adverse events
(21, 21)	USA	3	IBS-D	ROME III	39.1 (15.8) ^†^	36.2 (16.5) ^†^	30 (62.5%)	14 (61%)	16 (64%)	236 (95)	221 (105)	Decrease in IBS-SSS ≥ 50 points at 3 months	Change in IBS-QoL, HADS, Bristol stool scale scores, microbiome profile, and adverse events
(45, 17)	Norway	1	IBS-C 38.2%, IBS-D 39.1%, IBS-M 22.7%	ROME IV	39.2 (12.4)	41.2 (13.7)	23 (21.1)	14 (25.9)	8 (14.8)	311.8 (76.8)	315.2 (77.1)	Decrease of IBS-SSS ≥ 50 points at 3 months	Reduction in the dysbiosis index (DI) and change in microbiome profile
(45, 17)	Norway	1	IBS-C 38.5%, IBS-D 37.6%, IBS-M 23.9%	ROME IV	39.3 (13.2)	41.2 (13.7)	17(15.5)	9 (16.4)	8 (14.5)	313.9 (87.3)	315.2 (77.1)	Decrease of IBS-SSS ≥ 50 points at 3 months	Reduction in the dysbiosis index (DI) and change in microbiome profile
(20, 20)	Denmark	2	IBS-C 33.3%, IBS-D 29.4%, IBS-M 37.3%	ROME III	37.3 (12.5)37.3 (12.5)37.3 (12.5)	35.5 (10.6)	16 (31.4%)	8 (32%)	8 (30.8%)	341.68 (95.02)	345.04 (79.56)	Decrease in IBS-SSS ≥ 50 points at 3 months	Change in IBS-QoL at 3 months and change in microbiome diversity
(38, 38)	Sweden	1	IBS-C 25%, IBS-D 56.25%, IBS-M 18.75%	ROME III	37.0 (19.7) ^†^	38.3 (8.9) ^†^	8 (50%)	5 (62.5%)	3 (37.5%)	NR	NR	The effect of FMT on the change of GSRS-IBS within 6-month follow-up period.	IBS-SSS, their general health and quality of life (SF-36 and IBS QoL), hospital anxiety and depression scale, visceral perception, and microbiome profile
(18, 18)	Belgium	1	IBS-D, IBS-M	ROME III	40.4 (7.8) ^†^	37.4 (12.2) ^†^	24 (38.7%)	13 (31%)	11 (59%)	370 (310–440)	NR	Self-reported adequate relief of general IBS symptoms and abdominal bloating at 3 months	Changes in IBS symptom scores and IBS- related quality of life
(19)	Norway	1	IBS-D 53%, IBS-M 47%	ROME III	43.6 (16.0)^†^43.6 (16.0)^†^	45.3 (18.0)^†^	55 (66.3%)	19 (34.5%)	9 (32.1%)	260 (226–313)	278 (223–254)	Decrease in IBS-SSS > 75 points at 3 months	Decrease in IBS-SSS > 75 points at 12 months
(39, 39)	Finland	4	IBS-D 51%, IBS-M 14.3%, IBS-U 28.6%, other6.1%	ROME III	47.3 (16.8)	46.3 (14.3)	20 (40.8%)	11 (47.8%)	9 (34.6%)	282.5 (85.4)	263.5 (93.2)	50-point decrease in the IBS-SSS total score as compared to the baseline throughout the 52-week follow-up period	Mental health including depression and anxiety, quality of life, microbiota change, and adverse events

NR, not reported; IBS, irritable bowel syndrome; IBS-SSS, IBS Severity Scoring System; IBS-QoL, IBS quality of life; GSRS-IBS; Gastrointestinal Symptom Rating Scale-IBS; FMT, fecal microbiota transplantation.

^†^Estimating of mean and SD using statistical technique by Luo D et al. Stat Methods Med Res. 2018;27(6):1785-1805.

We performed a meta-analysis of 7 studies with 9 randomized control trials involving FMT in patients with IBS. All patients in these studies were adults. Five studies (18–20, 38, 39) included only a placebo and an intervention group. The El Salhy et al.’s study (17), included a placebo group and 2 intervention groups: FMT 30 g and FMT 60 g and the Aroniadis et al.’ study (21) conducted a cross-over trial. Therefore, there were 9 included cohorts for analysis. The placebo was the autologous stool if FMT was performed by an endoscopic technique and was placebo capsules if FMT was performed *via* oral capsules.

### Global symptom outcomes

There were 7 (17–21, 38, 39) studies (9 cohorts) and 2 studies (19, 39) that reported short- and long-term global symptom outcomes, respectively. The primary outcome analysis comprised all 505 patients, of who 302 received FMT and 203 received a placebo. At 12 weeks, the global improvement of IBS symptoms was 57% (201/302) in patients randomized to donor FMT and 42.9% (87/203) in patients given to placebo.

**FIGURE 2:**
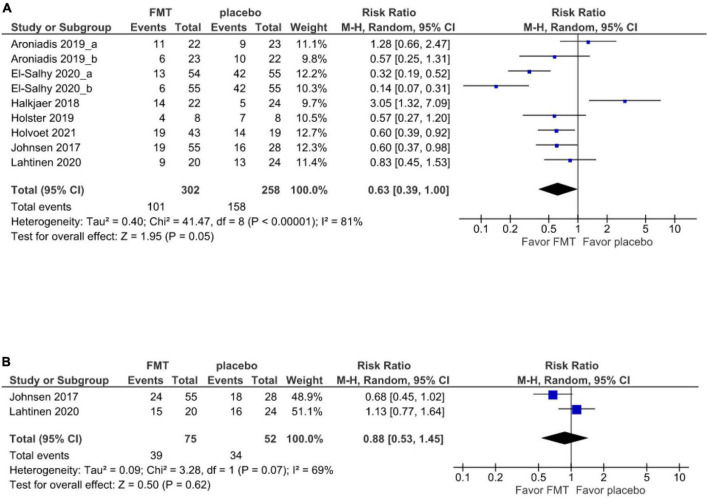
Forest plot of global symptom of IBS between FMT and placebo. **(A)** Short term. **(B)** Long term.

There was no significant difference in the global improvement of IBS symptoms between patients receiving donor FMT and those receiving placebo at 12 weeks (RR 0.63, 95% CI 0.39–1.00, *I*^2^ 81%) (Figure 2A).

At 52 weeks, the 2 studies (19, 39) report that the global improvement of IBS symptoms was 48% (36/75) in patients randomized to donor FMT and 34.6% (18/52) in patients given a placebo. However, there was no significant difference in the global symptom improvement compared between the FMT and placebo groups at 52 weeks (RR 0.88, 95% CI 0.53–1.45, *I*^2^ 69%) (Figure 2B).

### Irritable bowel syndrome symptom severity outcomes

**FIGURE 3:**
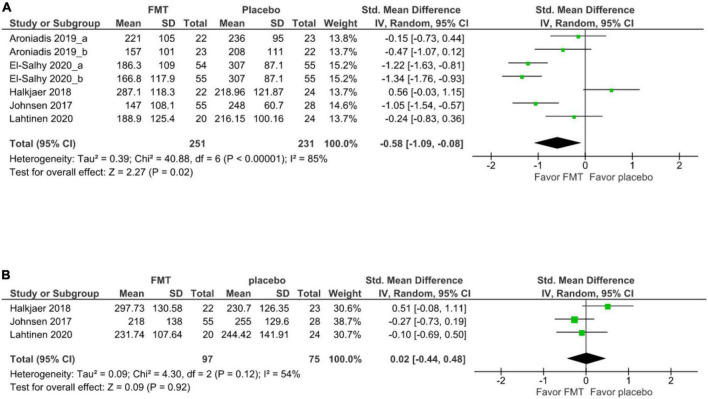
Forest plot of IBS-SSS outcome between FMT and placebo. **(A)** Short term. **(B)** Long term.

For secondary outcomes, 5 studies (7 cohorts) (21, 17, 20, 19, 39) evaluated the short-term improvement of IBS-SSS. There was significant improvement of IBS-SSS after FMT relative to after placebo at 12 weeks (SMD –0.58, 95% CI –1.09 to –0.88, *I*^2^ 85%) (Figure 3A). The mean difference in the improvement of IBS-SSS between FMT and placebo at 12 weeks was –60.54 with a 95% CI was -108.73 to –12.34.

Three studies evaluated the improvement of IBS-SSS between FMT and placebo at 52 weeks (19, 20, 39). There was no significant difference in IBS-SSS between patients receiving donor FMT and those receiving (SMD 0.02, 95% CI –0.44–0.48, *I*^2^ 54%) (Figure 3B).

### Quality of life

**FIGURE 4:**
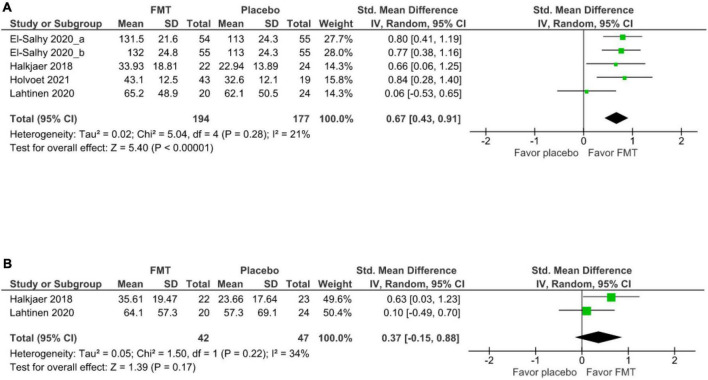
Forest plot of IBS-QoL outcome between FMT and placebo. **(A)** Short term. **(B)** Long term.

Four studies (5 cohorts) (17, 20, 18, 39) evaluated the short-term improvement of IBS-QoL between FMT and placebo groups at 12 weeks, FMT significantly improved IBS-QOL relative to placebo (SMD 0.67, 95% CI 0.43–0.91, *I*^2^ 21%) (Figure 4A). Two studies (20, 39) demonstrated that there was no significant difference of long-term IBS-QoL between FMT and placebo groups at 52 weeks (SMD 0.37, 95% CI –0.15–0.88, *I*^2^ 34%) (Figure 4B).

### Subgroup analysis of primary outcome

#### Association of the risk of bias and clinical outcomes

Among 6 studies (8 cohorts) (17–19, 21, 38, 39) with low-risk bias, 6 studies (8 cohorts) (17–19, 21, 38, 39) and 2 (19, 39) evaluated short and long-term global symptom improvement, respectively. There was significant improvement of short-term global symptoms between patients receiving FMT and those receiving placebo (RR 0.53, 95% CI 0.35–0.81, *I*^2^ 74%) (Supplementary Figure 1). However, there was no significant difference in the long-term global symptom improvement compared between the FMT and placebo (RR 0.88, 95% CI 0.53–1.45, *I*^2^ 69%) (Supplementary Figure 2A).

#### The characteristics of stool donors and clinical outcomes

Six studies (7 cohorts) (17–20, 38, 39) described inclusion and exclusion criteria of the donors. The inclusion and exclusion criteria of stool donors were described in Supplementary Table 3.

### Studies with well-defined donors

Among 2 studies (3 cohorts) (17, 18) with well-defined donors, all of these studies (17, 18) and none of these studies evaluated short and long-term global symptom improvement, respectively. There was significant difference in the short-term global symptom improvement compared between the FMT and placebo (RR 0.31, 95% CI 0.14–0.72, *I*^2^ 85%) (Supplementary Figure 3). The characteristics of well-defined stool donors were demonstrated in Table 3.

**TABLE 3:** The characteristics of stool donor.

	Author, year	Number of donors	Sex	Exclusion criteria of donors	Donor standardization		Factor that positively influence microbiome diversity								
					Maintain long term behavior	Stability of microbiome composition over time	Specific characteristics								Microbiome diversity
							Normal BMI	Age < 50 year	Born vaginally	Non-smoker	Regular exercise	Without frequent treatment with antibiotics	Consuming a sport-specific diet	Without first degree relative	
Well-defined donor	(45, 17)	1	Male	Yes	Prohibited from taking medication	Yes	Yes	Yes	Yes	Yes	Yes	Yes	Yes	Yes	A significant degree of microbial diversity (normobiotic) with bacterial signature including an abundance of *Streptococcus, Dorea, Lactobacillus*, and *Ruminococcaceae* spp. These four genera of bacteria have been reported to constitute favorable bacteria for a donor.
	(45, 17)	1	Male	Yes	Prohibited from taking medication	Yes	Yes	Yes	Yes	Yes	Yes	Yes	Yes	Yes	A significant degree of microbial diversity (normobiotic) with bacterial signature including an abundance of *Streptococcus, Dorea, Lactobacillus*, and *Ruminococcaceae* spp. These four genera of bacteria have been reported to constitute favorable bacteria for a donor.
	(18, 18)	2	Male	Yes	Prohibited from taking medication	Yes	Yes	Yes	N/A	N/A	N/A	N/A	N/A	N/A	Two donors had high microbial diversity
				and travelling abroad. Were requested to maintain a stable diet during the study.											
Relatively well-defined donor	(20, 20)	4	N/A	Yes	Informed to maintain a healthy lifestyle throughout the collection period	N/A	Yes	Yes	Yes	Yes	N/A	N/A	N/A	N/A	Donors had higher microbiome biodiversity than patients with IBS
	(38, 38)	2	N/A	Yes	Asked to keep their medication and diet stable over the entire study period.	N/A	N/A	N/A	N/A	N/A	N/A	N/A	N/A	N/A	The subject with the highest abundance of the butyryl-CoA CoA transferase gene in their fecal sample was selected as donor.
	(39, 39)	1	Male	Yes	Prohibited from taking medication and traveling abroad	N/A	Yes	Yes	Yes	Yes	N/A	Yes	N/A	N/A	Donors had higher microbiome richness than patients with IBS
Unclearly defined donor	(21, 21)	4	N/A	N/A	N/A	N/A	Yes	Yes	N/A	N/A	N/A	N/A	N/A	N/A	N/A
	(21, 21)	4	N/A	N/A	N/A	N/A	Yes	Yes	N/A	N/A	N/A	N/A	N/A	N/A	N/A
	(19)	2	N/A	Yes	N/A	N/A	Yes	N/A	N/A	N/A	N/A	N/A	N/A	N/A	N/A

N/A. Not available; BMI, Body mass index.

### Studies with relatively well-defined donors

Among 3 studies (20, 38, 39) with relatively well-defined donors, all of these studies (20, 38, 39) evaluated a short-term and 1 study (39) evaluated a long-term global symptom improvement. There was no significant difference in the short-term global symptom improvement compared between the FMT and placebo (RR 1.10, 95% CI 0.43–2.82, *I*^2^ 80%) (Supplementary Figure 3). The characteristics of relatively well-defined stool donors were demonstrated in Table 3.

### Studies with unclearly defined donors

Among 2 studies (3 cohorts) (19, 21) with unclearly defined donors, all of these studies evaluated a short-term and 1 study (19) evaluated a long-term global symptom improvement. There was no significant difference in the short-term global symptom improvement compared between the FMT and placebo (RR 0.76, 95% CI 0.46–1.25, *I*^2^ 45%) (Supplementary Figure 3). The characteristics of the unclearly defined donor were demonstrated in Table 3.

#### Association of fecal administration routes and clinical outcomes

Three studies (19, 38, 39) performed FMT using colonoscopy which 3 (19, 38, 39) and 2 (19, 39) of these studies evaluated short and long-term global symptom improvement, respectively. There was significant difference in the short-term global symptom improvement compared between the FMT and placebo (RR 0.66, 95% CI 0.47–0.92, *I*^2^ 0%) (Supplementary Figure 4). However, there was no significant difference in the long-term global symptom improvement compared between the FMT and placebo (RR 0.88, 95% CI 0.53–1.45, *I*^2^ 69%) (Supplementary Figure 2B).

Four studies (6 cohorts) (17, 18, 20, 21) performed FMT using upper gut administration which 2 studies (3 cohorts) (21, 20) used FMT capsules, 1 study (2 cohorts) (17) used Gastroscopy, and 1 study (18) used nasojejunal probe. All of these studies (17, 18, 20, 21) evaluated only short-term but not long-term global symptom improvement. There was no significant difference in the short-term global symptom improvement compared between the FMT and placebo (RR 0.62, 95% CI 0.30–1.28, *I*^2^ 88%) (Supplementary Figure 5A).

Two studies (3 cohorts) (21, 20) in which patients received FMT *via* capsule evaluated only a short-term but not a long-term global symptom improvement. There was no significant difference in the short-term global symptom improvement compared between the FMT and placebo (RR 1.30, 95% CI 0.54–3.14, *I*^2^ 74%) (Supplementary Figure 4).

#### Association of types of feces and clinical outcomes

Five studies (7 cohorts) (17, 20, 21, 38, 39), 1 study (18), and 1 study (19) used frozen feces, fresh feces, and both as types of feces in FMT, respectively. Subgroup analysis of the 5 studies (7 cohorts) (17, 20, 21, 38, 39) using frozen stools showed non-significant results of short-term global symptom improvement (RR 0.64, 95% CI 0.32–1.27, *I*^2^ 86%) (Supplementary Figure 5B). Lahtinen et al. (39) evaluated and showed non-significant long-term global symptom improvement in comparison between the FMT and placebo (RR 0.83, 95% CI 0.45–1.53). Holvoet et al. using fresh stool (18) revealed significant difference in the short-term global symptom improvement compared between the FMT and placebo (*p* = 0.03). Johnsen et al. (19) used both types of feces but did not report outcomes of short-term global symptom improvement by types of feces separately.

#### Microbiome profiles and clinical outcomes

Six studies (8 cohorts) (17, 18, 20, 38, 39) reported microbiota analysis. Five studies (7 cohorts) (17, 18, 20, 21, 38) reported on microbiome diversity and 1 study (39) reported microbiome richness. The 6 studies (8 cohorts) (17, 18, 20, 21, 38, 39) explored microbiota in stool and 1 study (38) explored both microbiota in stool and microbiota at the mucosa. The microbiome profile was assessed in both donors and recipients among 422 patients in 6 studies. Our study focused on 2 aspects of the gut microbiome, including the donors’ microbiota vs. patients’ microbiome profile and changes of patients’ microbiota after FMT.

### Baseline donors’ microbiota vs. baseline patients’ microbiota

Six studies (8 cohorts) (17, 18, 20, 21, 38, 39) provided baseline donor microbiome information and baseline patient’s microbiome. Three studies (4 cohorts) (17, 18, 20) reported that donors’ microbiome diversity was higher than patients’ microbiome diversity at baseline. These studies evaluated only a short-term global symptom improvement and there was no significant improvement in short-term global symptoms (RR 0.53, 95% CI 0.20–1.41, *I*^2^ 91%) (Supplementary Figure 5C). One study (38) in which donor microbiome diversity was different from patient microbiome diversity at baseline could not demonstrate a significant improvement in short-term global symptoms (RR 0.57, 95% CI 0.27–1.20). Also, one study (39) in which donors’ microbiome richness was higher than baseline patients’ microbiome richness, could not demonstrate a significant improvement in short-term global symptoms (RR 0.83, 95% CI 0.45–1.53). Finally, 1 study (2 cohorts) (21) did not report association between donors’ and patients’ microbiome diversity at baseline. Comparison between the donor and patient microbiome profile at baseline was shown in Supplementary Table 4.

Two studies (3 cohorts) (17, 20) evaluated the association between donor microbiome profile and clinical improvement after FMT, and 2 studies (3 cohorts) (18, 21) evaluated the association between patient microbiome profile at baseline and clinical improvement after FMT. Two studies (4 cohorts) (17, 21) demonstrated that clinical improvement was associated with specific bacteria in the donor and patient microbiome, respectively, while another 2 studies (18, 20) demonstrated that clinical improvement was not associated with donor and patient microbiome diversity at baseline, respectively. The association between microbiome profile and clinical symptom improvement was shown in Supplementary Table 5.

### Changes of patients’ microbiota after fecal microbiota transplantation

Five studies (7 cohorts) (17, 20, 21, 38, 39) reported information about short-term changes of patients’ microbiota after FMT, and one study (39) reported information about long-term changes of patients’ microbiota after FMT. However, the findings from each study were not coherent. There was only one study (20) demonstrating that patients had a significant increase in microbiome diversity shift to the donor after FMT. One study (38) showed that patients had an increase in microbiome diversity shift to the donor after FMT, but no statistical significance. One study (2 cohorts) (21) revealed that patients’ microbiome diversity after FMT shifted to the donor; however, the study did not specify whether the patient’s microbiome diversity after FMT was increased or decreased. One study (2 cohorts) (17) found no significant change in patient microbiome diversity. The other study (39) reported that microbiome richness shifted to the donor after FMT; however, the increase in microbial richness was not reflected as an increase in the microbial diversity. Thus, this information was insufficient to conduct subgroup analysis in comparison between patients whose gut microbiome diversity changed following FMT and patients whose gut microbiome diversity did not change following FMT. Comparison between the donor and patient microbiome profile after FMT was shown in Supplementary Table 4.

Three studies (4 cohorts) (17, 20, 38) demonstrated the association between patient microbiome profile after FMT and clinical improvement of IBS. Two studies (3 cohorts) (17, 20) reported the specific bacteria associated with IBS-SSS score. One study (38) demonstrated that patient microbiome profiles after FMT were not significantly associated with global symptom improvement. The association between microbiome profiles and clinical symptom improvement was shown in Supplementary Table 5.

### Safety and adverse effects

Six studies with 8 cohorts (17, 19, 20, 21, 38, 39) with 270 FMT patients had their short-term adverse event data reviewed. Most adverse events are minor, self-limiting, and occur during the first few days after transplantation, including diarrhea (14.4%), abdominal pain or cramping (13.0%), and constipation (10.4%). One severe adverse event of transient vertigo and nausea occurred after the FMT, necessitating a few hours of hospital monitoring was reported (19).

### Publication bias

According to Cochrane guidelines, one study was considered having an uncertain risk of bias because it did not adequately explain allocation concealment (Supplementary Figure 6). A funnel plot was used to illustrate the dispersion and heterogeneity of the research considered. Our calculations of *I*^2^-value show that the distributions of the included studies were heterogeneity. The Egger regression test did not revealed data asymmetry (*P*-value = 0.5091). The funnel plot was shown in Supplementary Figure 7.

## Discussion

This systematic review and meta-analysis evaluated the efficacy of FMT in the treatment of IBS and conducted subgroup analyses to determine the factors that influence the efficacy of FMT in IBS. Recently, the meta-analyses of RCTs regarding FMT and IBS were published in the year 2022 (23, 24). Consistent with these meta-analyses, we found that FMT does not result in a significant global improvement in IBS patients, but that FMT performed *via* colonoscopy significantly improved global IBS symptoms. However, our meta-analysis provides additional results that the prior meta-analysis did not. First, our meta-analysis is the first to find a significant improvement in IBS-SSS after FMT compared to placebo after 12 weeks. The result was different from previous meta-analyses since the El-Salhy et al. study (17) reported more than one method of intervention (30 g FMT and 60 g FMT), we extracted this study into two separate “cohorts” per Cochrane’s recommendation (40). Also, these two cohorts show a significant improvement in global IBS symptoms in the FMT group compared to the placebo group. Second, our meta-analysis is the first to conduct the subgroup analysis of low-risk of bias and found a significant improvement of the global symptom of IBS after FMT. Third, we established the criteria for stool donor (well-defined, relatively well-defined and unclearly defined donor). In addition, we conducted a subgroup analysis of stool donors and discovered that a well-defined donor subgroup had significant global symptom improvement of IBS after FMT. Well-defined donors with specific microbiome profile may need to be chosen as the perfect stool donor for FMT in IBS. Finally, since microbiome profile is one of the important factors influencing the efficacy of FMT and IBS, we compared the microbiome profile (the baseline donor microbiome profile, the baseline patient microbiome profile, and the patient microbiome profile after FMT) and global IBS symptom improvement.

We discovered that the mean difference in IBS-SSS scores between the FMT and placebo groups was –60.54. IBS-SSS has a total score of 500 points. Thus, IBS-SSS was improved by approximately 12% in FMT compared with placebo. However, the proposed definition of patients with improved global symptoms in IBS were improving by 30% in IBS-SSS score (38, 41). This could explain why there was no statistically significant improvement of global symptoms despite statistically significant differences in short-term IBS-SSS improvement.

Although FMT significantly improved IBS-SSS and IBS-QoL in IBS patients over the short-term, the long-term results were insignificant. It has been observed that FMT has substantial effects on the first day after administration (42); however, the decrease of donor strain populations has been observed 1.5–3 months after FMT (43). In tandem with the decrease of donor strains, the theoretical efficacy of FMT will decline substantially (43). Thus, repetitive FMT may be necessary (44). El-Salhy et al. (45) demonstrated that patients with IBS who were unresponsive to 30 g FMT repeated the 60 g FMT 3–4 months after the first FMT. 70% of these individuals had significant clinical improvements in abdominal symptoms, fatigue, and QoL in 57, 80, and 67%, respectively. In addition, Cui et al. (44) demonstrated that the responsiveness diminished over time after the FMT treatment period. Also, FMT should be repeated every 3 months in ulcerative colitis (46). Therefore, repeated and periodic FMT for IBS may significantly maintain and improve FMT’s efficacy. Although some previous studies demonstrated appropriate time for repeated FMT are 3–4 months, additional RCTs should be conducted to determine the exact optimal duration for repetitive FMT.

While statistical significance of the included studies in the improvement of global symptoms was not found, the subgroup analysis of the studies with the low risk of bias (17, 18, 19, 21, 38, 39) demonstrated significant short-term global IBS symptom improvement. From all included studies, Halkjær et al. (20) was the only study that demonstrated an unclear risk of bias due to the absence of allocation concealment information and was excluded from the subgroup analysis. The plausibility that led overall pooled results to non-significance could be the true effect of FMT that did not improve the global symptoms of IBS, the route of administration, which was mentioned below as one key factor that may contribute to the favor-placebo result, the heterogeneity, and the absence of an allocation concealment process. The significant result of low-risk bias studies supports that the true effect of FMT improving the global symptoms of IBS and additional high-quality RCTs with an unbiased randomization process will be likely to demonstrate a significant global improvement in IBS symptoms.

Subgroup analyses of well-defined donor subgroups (17, 18) were performed because perfect stool donors may be essential for FMT in resolving IBS (47). Fecal microbiota has an indirect role in the development and treatment of IBS (through bile acid and short-chain fatty acid metabolism) and is affected by host-associated variables. This may be considered donor dependence (47). We can infer from the Holvoet et al. study (18) that along with high diversity, the stability of the donor’s microbial composition may be a significant predictor of success. Therefore, we should follow donors’ microbiomes for a long period of time to determine the stability of their microbial composition. Moreover, we can infer from the El-Salhy et al. study (17) that characteristics that had a beneficial effect on gut microbiota were essential in improving global symptoms, such as few antibiotic consumptions, regular physical activity, breastfeeding, delivery *via* normal labor, no smoking and few antibiotics. Also, the donor was normobiotic with a bacterial signature that included an abundance of *Streptococcus, Dorea, Lactobacillus*, and *Ruminococcaceae spp*. which have been reported to constitute favorable bacteria (17). Probiotics from *Lactobacillus* and *Streptococcus* spp. showed a trend toward symptomatic improvement in IBS patients (48). Moreover, *Ruminococcaceae, Dorea, and Lactobacillus* spp. have been identified as Butyrate-producing bacteria (49–51). Additional research with well-defined donors is required to identify favorable bacterial signatures especially butyrate-producing bacteria as the specific bacteria that will be chosen in the future as the perfect stool donor.

Although we defined the other studies as relatively well-defined and unclearly defined donors, we cannot conclude that these studies did not assess microbial diversity and stability adequately. Some studies including Holster et al. (38) and Lahtinen et al. (39) did not conclude the stability of the microbial composition; however, they measured the microbiome diversity throughout the duration of the study and discovered that the donor microbiome diversity were higher diversity than patient microbiome diversity. Therefore, we can infer that these donors may have stable microbial composition although there is no direct assessment to support this conclusion.

Apart from being perfect stool donor, patients’ microbiome profiles may be one of the important factors of the clinical improvement of IBS symptoms after FMT. In our meta-analysis, the genus *Prevotella* was detected in high levels in FMT responders (21). Moreover, participants with higher levels of *Lactobacillus spp*. concentrations and *Blautia* genus of the *Clostridiales* order respond better to treatment or have a greater reduction in IBS-SSS score (17, 20). These 3 genera of bacteria have been identified as butyrate-producing bacteria which are the favorable bacteria (13, 51–55). Additional high-quality RCTs will be necessary to identify specific favorable bacteria especially butyrate-producing bacteria in patient groups that may serve as a good predictor of clinical improvement in IBS symptoms.

Subgroup analysis of the studies (17, 18, 20) in which donor microbiome diversity was higher than that of patients at baseline demonstrated a non-significant improvement in global symptoms because the Halkjær et al. study (20) found no improvement in global symptoms in the FMT group. The reason why Halkjær et al. showed no improvement in global symptoms in the FMT group could be related to the route of administration of FMT capsules. Oral administration of fecal bacteria to the upper GI tract may inadvertently exacerbate underlying functional GI symptoms (56). On the other hand, subgroup analysis of the studies using colonoscopy as the route of administration demonstrated statistically significant improvement in global IBS symptoms. According to one meta-analysis (57), *Clostridioides difficile* infection cure rates with FMT administered through colonoscopy are superior to those with enema and nasogastric tube. Moreover, patients who get FMT through colonoscopy had a lower rate of remission than those who receive FMT *via* upper gastrointestinal infusions in inflammatory bowel disease (58). The reason colonoscopy-guided FMT seems to be efficacious may be that most patients treated with colonoscopy-guided FMT received pre-FMT lavage and a larger volume of stool suspension infusion per FMT, both of which may have contributed to the effectiveness results. Therefore, administering FMT (59) to IBS patients *via* colonoscopy may be the most effective method, while administering *via* FMT capsule may not be the good option.

Subgroup analysis of frozen stools as a type of feces demonstrated no statistical significance of short-term improvement in global IBS symptoms. Whereas Holvoet et al. who used fresh stool reported significant short-term improvement in global IBS symptoms. However, among the studies with frozen feces, 2 studies (3 cohorts) (20, 21) *via* FMT capsule, 2 studies (38, 39) were conducted *via* colonoscopy, and 1 study (17) *via* gastroscopy. As the route of administration could impact the results, the conclusion that using frozen stools is inferior to fresh stools cannot be drawn in FMT of patients with IBS from this study. Recently, there were no studies that compared FMT *via* frozen stools with fresh stools among IBS patients. Lee et al. (60) conducted a non-inferiority trial of FMT with frozen stools in comparison with fresh stools for recurrent *Clostridioides difficile* infection and showed the non-inferiority of FMT using frozen compared with fresh feces on clinical resolution rates. Moreover, using frozen stools from universal donors could reduce costs and time used in the preparation (61, 62). Additional RCTs compared the efficacy of types of feces on FMT in IBS patients are needed to conclude the preferable type of feces.

There are limitations to this systematic review and meta-analysis. First, as the number of identified studies was fewer than 10, the Egger’s regression asymmetry test and funnel plots did not effectively indicate only publication bias but a variety of possible reasons such as heterogeneity, chance, and publication bias (63). Second, although our meta-analysis revealed that FMT improved IBS-SSS score statistically significantly, it improved below the FDA-approved cutoff of 30% improvement in IBS-SSS to be considered as global symptom improvement. Therefore, FMT should not be recommended in clinical practice for IBS treatment until the FMT techniques are developed and provide the improvement of the IBS-SSS score by more than 30%. Third, although a well-defined donor may be associated with a favorable outcome, little is known about the favorable microbiome profile of such donors. It might be too early to determine an appropriate stool for FMT in IBS. Fourth, the assumption that FMT *via* colonoscopy can improve global IBS symptoms may be only partially correct. This could be because pre-FMT lavage and a higher volume of stool suspension infusion per FMT contribute to the FMT’s effectiveness. Fifth, only a few studies reported on the association between global symptom improvement and donor microbiome profile, the patient microbiome profile at baseline, and the patient microbiome profile following FMT. Therefore, the results in this meta-analysis about microbiome profile and global symptom improvement were limited. Sixth, the results of this meta-analysis should not be applied to pediatric patients, as only studies in adults were available for this meta-analysis. Seventh, Additional factors that may limit the generalizability of the results include the fact that all stool donors are men or N/A. In addition, all of the included studies was conducted in either Europe or the United States. Finally, statistical heterogeneity was high in this meta-analysis. The factors that contributed to the heterogeneity may be route of administration and risk of bias, respectively. When we performed subgroup analysis on these factors, heterogeneity was reduced. In particular, the I^2^ for the colonoscopy subgroup is 0. Therefore, additional RCTs with colonoscopy as the route of administration and a low risk of bias are required to reduce statistical heterogeneity in prospective meta-analyses. Also, high-quality RCTs with large sample sizes, well-defined donors, appropriate route of administration and well-studied gut microbiome profiles of donors & recipients are needed to confirm the efficacy of FMT in IBS.

## Conclusion

In conclusion, this meta-analysis of FMT in IBS demonstrated that FMT had a significant positive impact on the IBS-SSS and IBS-QoL in the short term, although its long-term efficacy was unclear. The improvements in clinical outcome with FMT for IBS may be attributed to the difference in route of administration, the donor selection criteria, and donor microbiome profile.

## Data availability statement

The original contributions presented in this study are included in the article/Supplementary material, further inquiries can be directed to the corresponding author.

## Author contributions

CS, RP, TP, and SG involved in the conception and design the study. CS, PK, and RP extracted the data and involved in the methodology. RP, TP, and SG involved in supervision and validation and critically revised the manuscript. CS and PK wrote the manuscript. All authors contributed to the article and approved the submitted version.

## Supplementary material

Supplementary Appendix 1 Search strategy.

Supplementary Figure 1 Subgroup analysis of risk of bias on improving global outcome in a short-term period.

Supplementary Figure 2 Subgroup analysis of **(A)** low risk of bias group and **(B)** route of colonoscopy FMT on improving global symptom outcomes in a long-term period.

Supplementary Figure 3 Subgroup analysis of type of stool donor on improving global outcome in a short-term period.

Supplementary Figure 4 Subgroup analysis of route of administration on improving global outcome in a short-term period.

Supplementary Figure 5 Subgroup analysis of **(A)** upper gut administration, **(B)** frozen feces, and **(C)** donor: higher microbiome diversity than the patient’s on improving global outcome in a short-term period.

Supplementary Figure 6 Risk of bias among the nine included randomized controlled trials of fecal microbiota transplantation for treating irritable bowel syndrome.

Supplementary Figure 7 Funnel plot showing the dispersion and heterogeneity of included studies.

## Abbreviations

AEs: Adverse events
CI: Confidence interval
FMT: Fecal microbiota transplantation
GSRS-IBS: Gastrointestinal Symptom Rating Scale for IBS
GI: Gastroenterology
IBS: Irritable bowel syndrome
IBS-SSS: The Irritable bowel syndrome Severity Scoring System instrument
IBS-QoL: The Irritable bowel syndrome quality of life instrument
RCTs: Randomized controlled trial
RR: Relative risk
SMD: Standard mean difference
QoL: Quality of life.
