# Supplementary material for: Fecal microbiota transplantation in irritable bowel syndrome: A meta-analysis of randomized controlled trials
Source: Front Med (Lausanne). 2022 Nov 3;9:1039284. doi: 10.3389/fmed.2022.1039284 (PMC9669599; doi:10.3389/fmed.2022.1039284)
Supplement: Supplementary Appendix 1 — Search strategy. [file Data_Sheet_1.docx]

| Concept | Search term |
| --- | --- |
| fecal  #1 | “faecal”[tiab] OR “fecal*”[tiab] OR “feces”[tiab] OR “faeces”[tiab] OR “stool*”[tiab] |
| Microbiota  #2 | “microbiota*”[tiab] OR “microbiom*”[tiab] OR “microbial”[tiab] OR “gastrointestinal microbiome”[tiab] OR “microflora”[tiab] OR “flora”[tiab] OR (“enteric”[tiab] AND “bacteria”[tiab]) |
| Transplantation  #3 | “transplant*”[tiab] OR “transfusion*”[tiab] OR “implant*”[tiab] OR “installation*”[tiab] OR “donor*”[tiab] OR “enema”[tiab] OR “infusion*”[tiab] OR “transfer*”[tiab] OR “FMT”[tiab] OR “bacteriotherap*”[tiab] |
| irritable bowel syndrome  #4 | “irritable bowel syndrome*”[tiab] OR “IBS”[tiab] OR (“irritable”[tiab] AND (“bowel”[tiab] OR “colon”[tiab])) OR ((“gastrointestinal”[tiab] OR “intestinal”[tiab] ) AND (“transit*”[tiab] OR “motilit*”[tiab] )) OR “visceral hyperalgesia”[tiab] OR (“functional”[tiab] AND (“colonic”[tiab] OR “bowel” [tiab] OR "gastrointestinal"[tiab]) AND (“disease*”[tiab] OR “disorder*”[tiab])) |
| #5 | #1 AND #3 |
| #6 | #2 AND #3 |
| #7 | #1 AND #2 AND #3 |
| #8 | #5 OR #6 OR #7 |
| #9 : Total | #8 AND #4 |

**Total**

Pubmed 646 papers

Embase 1,197 papers

Cochrane 141 papers

Ovid 605 papers

Duplicate 1191 papers

**Total 1398 papers**
