# Supplementary figures and images for: Fecal microbiota transplantation in irritable bowel syndrome: A meta-analysis of randomized controlled trials
Source: Front Med (Lausanne). 2022 Nov 3;9:1039284. doi: 10.3389/fmed.2022.1039284 (PMC9669599; doi:10.3389/fmed.2022.1039284)

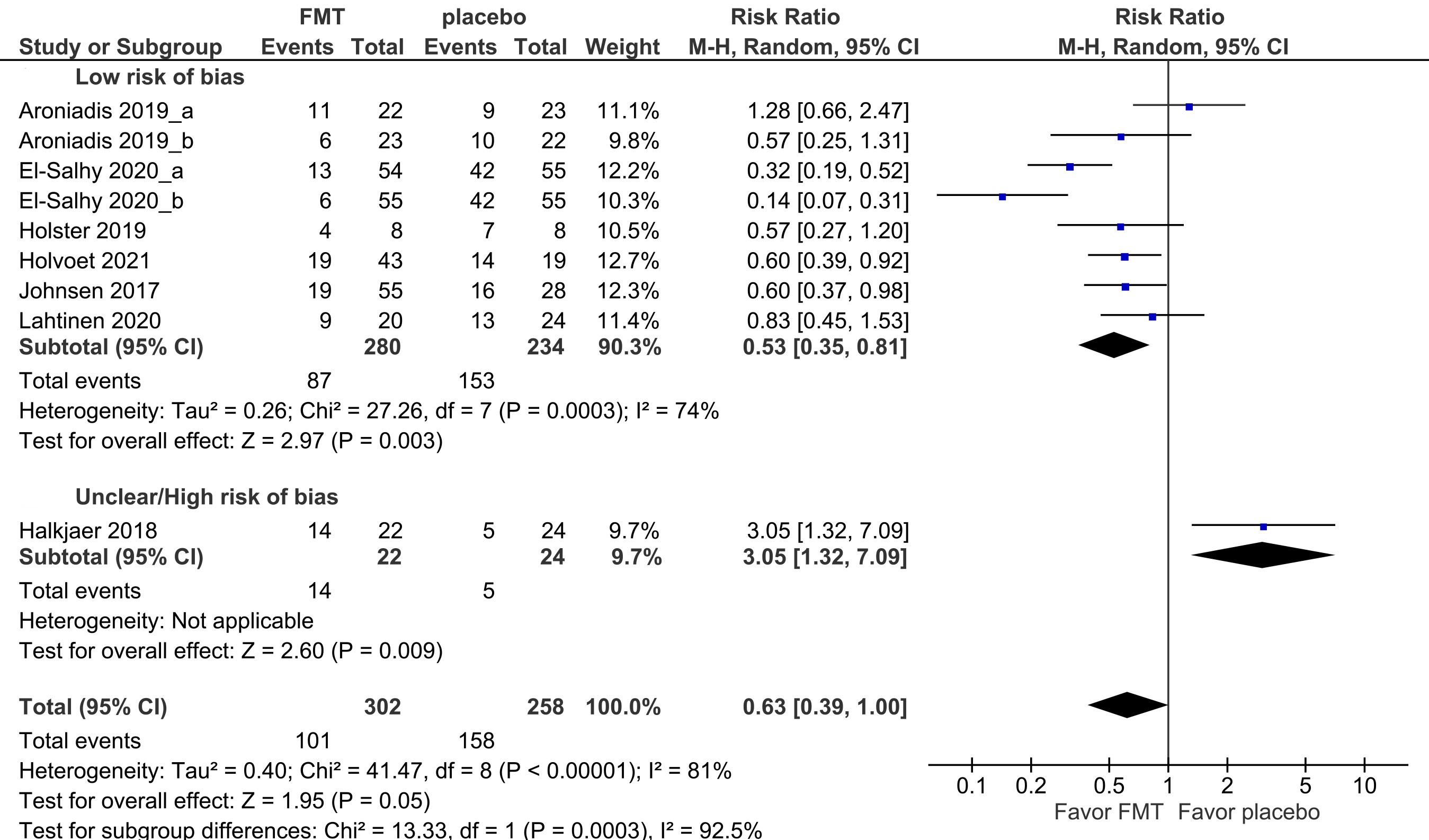

Supplement: Supplementary Figure 1 — Subgroup analysis of risk of bias on improving global outcome in a short-term period. [file Image_1.jpg]

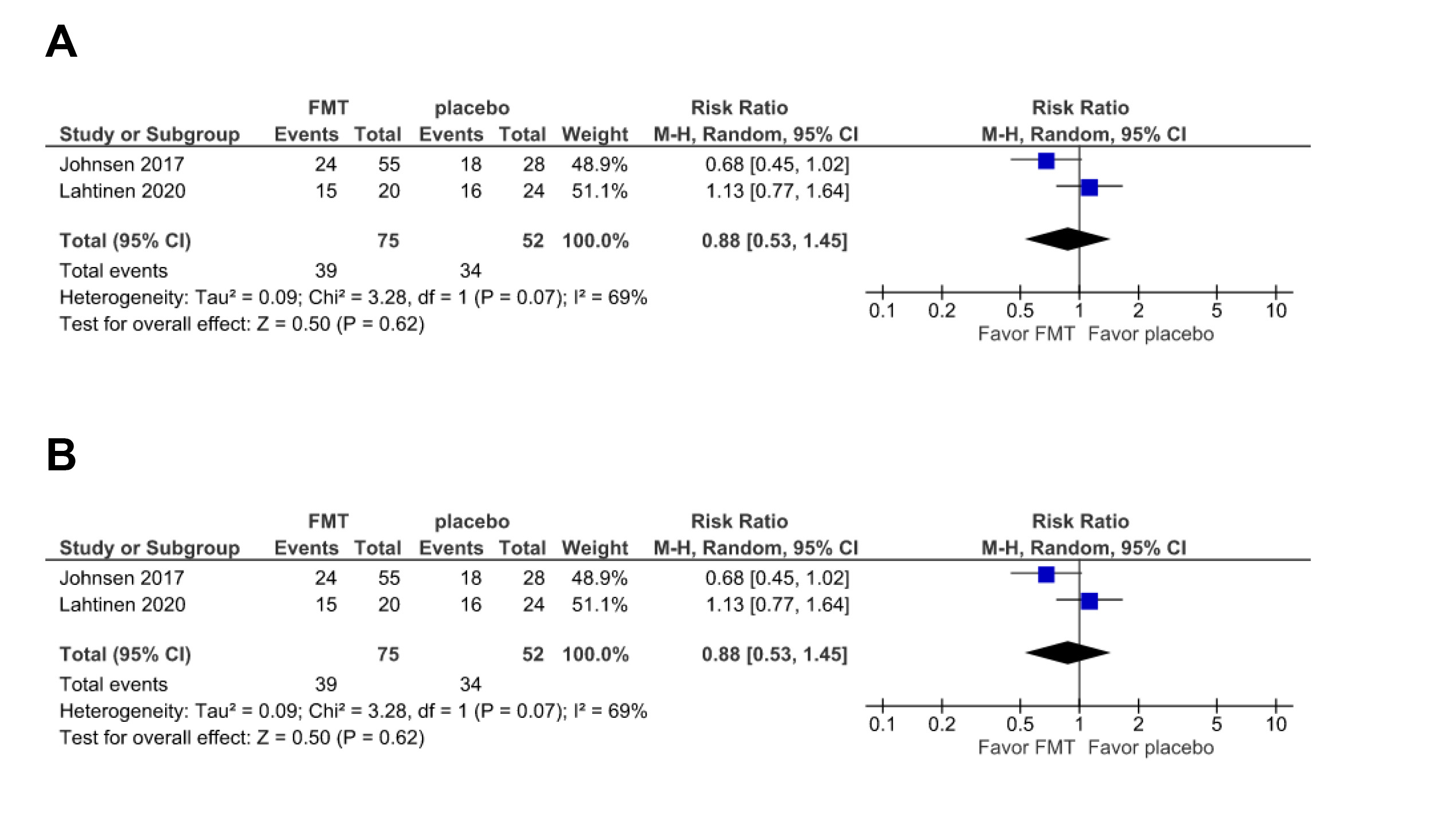

Supplement: Supplementary Figure 2 — Subgroup analysis of (A) low risk of bias group and (B) route of colonoscopy FMT on improving global symptom outcomes in a long-term period. [file Image_2.jpg]

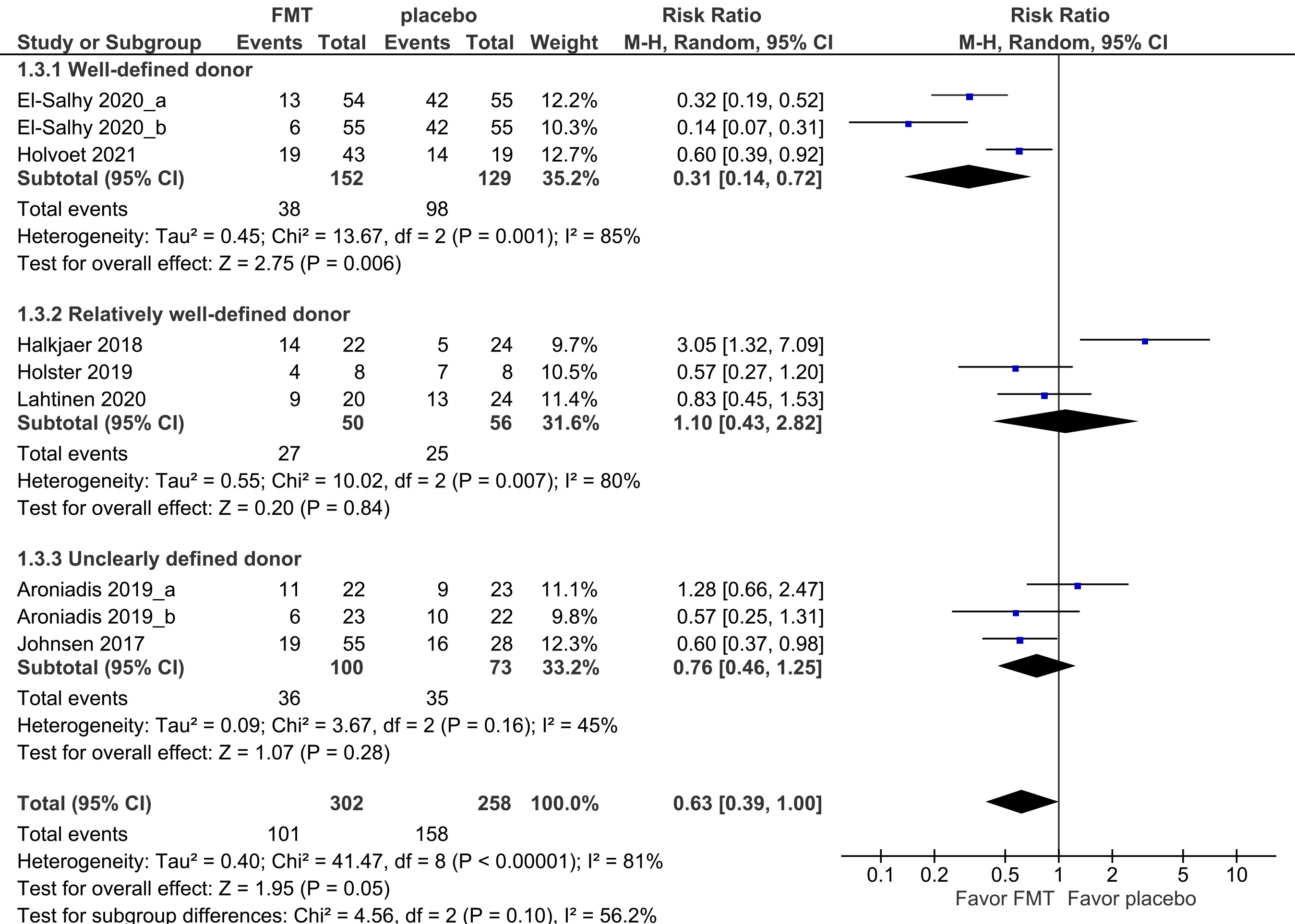

Supplement: Supplementary Figure 3 — Subgroup analysis of type of stool donor on improving global outcome in a short-term period. [file Image_3.jpeg]

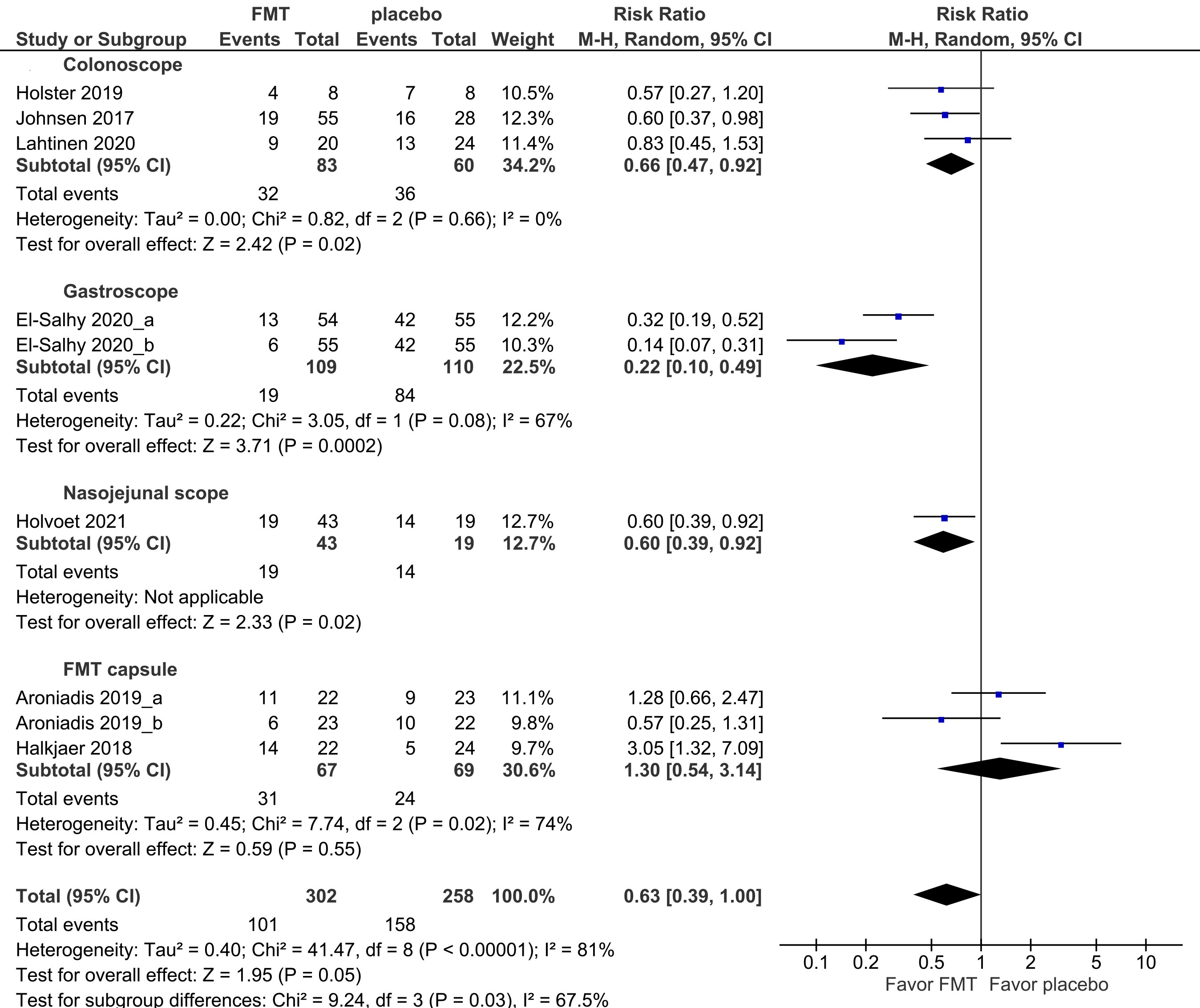

Supplement: Supplementary Figure 4 — Subgroup analysis of route of administration on improving global outcome in a short-term period. [file Image_4.jpg]

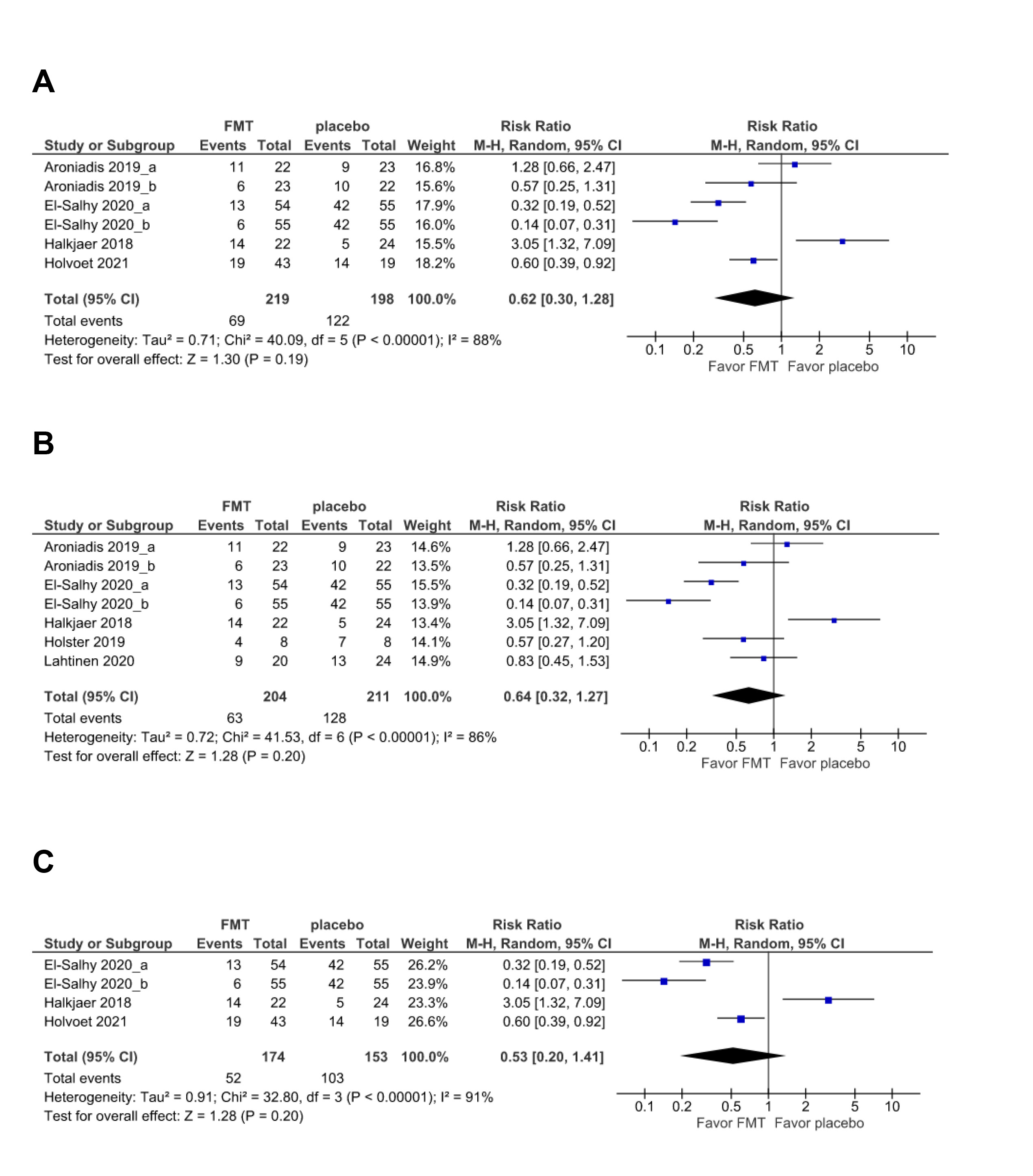

Supplement: Supplementary Figure 5 — Subgroup analysis of (A) upper gut administration, (B) frozen feces, and (C) donor: higher microbiome diversity than the patient’s on improving global outcome in a short-term period. [file Image_5.jpeg]

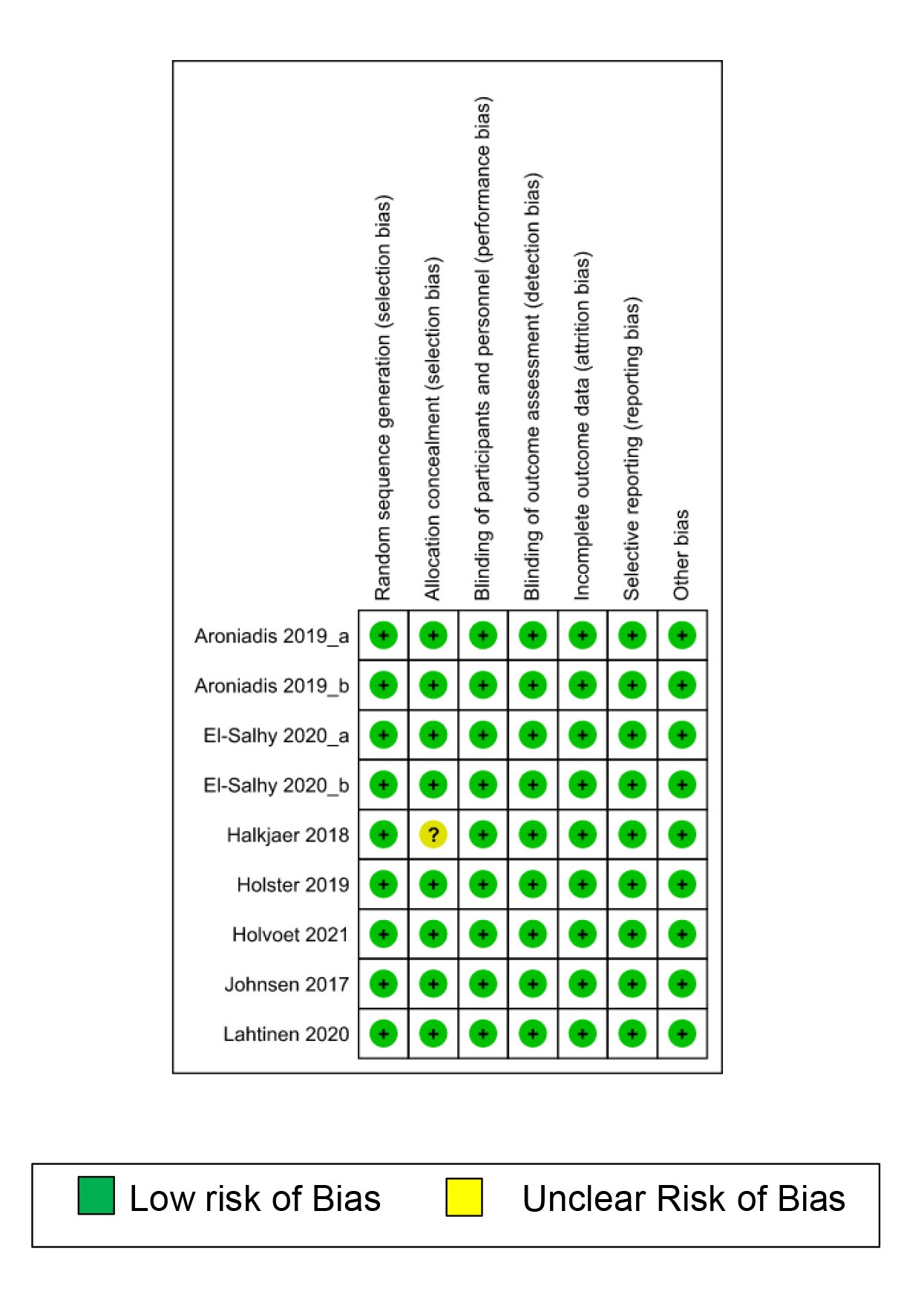

Supplement: Supplementary Figure 6 — Risk of bias among the nine included randomized controlled trials of fecal microbiota transplantation for treating irritable bowel syndrome. [file Image_6.jpeg]

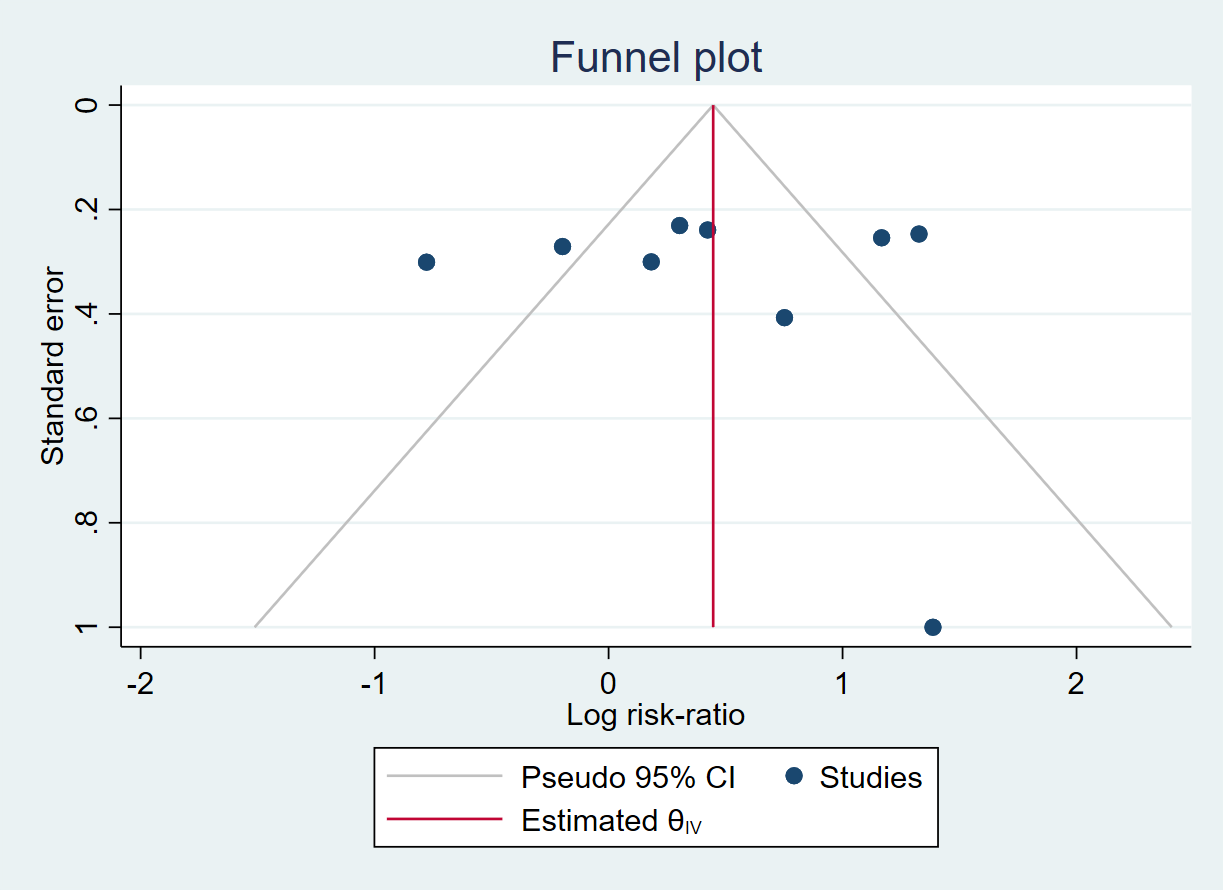

Supplement: Supplementary Figure 7 — Funnel plot showing the dispersion and heterogeneity of included studies. [file Image_7.jpeg]
