# Supplementary material for: Fecal microbiota transplantation in irritable bowel syndrome: A meta-analysis of randomized controlled trials
Source: Front Med (Lausanne). 2022 Nov 3;9:1039284. doi: 10.3389/fmed.2022.1039284 (PMC9669599; doi:10.3389/fmed.2022.1039284)
Supplement: Supplementary file 9 [file Table_1.pdf]

Supplementary Table 1: **Existing accepted protocols for donor screening** defined by Kim KO, Bakken JS, Brandt LJ and McCune VL

1.1 Suggested Exclusion Criteria for Stool Donors

**Exclusion criteria** *
Age <18 years or >65 years
BMI >30 kg/m^2
Metabolic syndrome
Moderate to severe undernutrition
History of antibiotics use in the last 6 months
Diarrhea within the last 3–6 months
History of *Clostridium difficile* colitis
Immune disorder or use of immunosuppressive medications
History of drug use or other recent risk factor for HIV or viral hepatitis
History of travel to a tropical region in last 3 months
Any gastrointestinal illness (inflammatory bowel disease, irritable bowel syndrome, gastrointestinal malignancy, or major surgery) or complaints
History of autoimmune or atopic illness
History of chronic pain syndrome (fibromyalgia, chronic fatigue syndrome)
Neurologic or neurodevelopmental disorders
History of malignancy

HIV, human immunodeficiency virus.
*Each institution can adopt different criteria.

1.2 Suggested Laboratory Tests for Stool Donor*

	Blood	Stool
Bacteria	- Treponema	- Enteric pathogen culture: *Salmonella, Shigella, Campylobacter*<br>- *Helicobacter pylori* EIA**<br>- Vancomycin-resistant Enterococcus
Viruses	- Hepatitis A virus IgM<br>- Hepatitis surface antigen<br>- Anti-hepatitis C virus<br>- HIV 1 and 2	- Norovirus EIA or PCR<br>- Rotavirus EIA
Parasites	- *Entamoeba histolytica*<br>- *Strongyloides stercoralis*	- Ovum and parasite<br>- Microsporidia<br>- *Giardia* fecal antigen/EIA<br>- *Cryptosporidium* EIA<br>- AFB for *Isospora* and *Cyclospora*
Others	- Complete blood count<br>- Liver function test<br>- ESR and CRP	- *Clostridium difficile* test<br>- Toxin PCR

AFB, acid-fast bacilli; CRP, C-reactive protein; EIA, enzyme immunoassay; ESR, erythrocyte sedimentation rate; HIV, human immunodeficiency virus; IgM, immunoglobulin M; PCR, polymerase chain reaction;.

*The blood and stool tests should be completed within 1 month of donation, and the tests could be adopted differently depending on each institution and circumstance.
**The test for *Helicobacter pylori* is usually needed in the case of upper gastrointestinal delivery.
