# Supplementary material for: Fecal microbiota transplantation in irritable bowel syndrome: A meta-analysis of randomized controlled trials
Source: Front Med (Lausanne). 2022 Nov 3;9:1039284. doi: 10.3389/fmed.2022.1039284 (PMC9669599; doi:10.3389/fmed.2022.1039284)
Supplement: Supplementary file 10 [file Table_2.pdf]

Supplementary Table 2: Summary of the descriptive characteristics of the included studies

Author, year	IBS- Patients						
	Year with IBS		Diet control	Current medication		Conventional therapy	
	FMT	Placebo		FMT	Placebo	FMT	Placebo
Aroniadis_a, 2019	NR	NR	NR	NR	NR	Antimotility drugs 8 (32%) Antispasmodic drugs 1 (4%) Antidepressants 3 (12%)	Antimotility drugs 11 (48%), Antispasmodic drugs 1 (4%) Antidepressants 2 (9%)
Aroniadis_b, 2019	NR	NR	NR	NR	NR	Antimotility drugs 11 (48%), Antispasmodic drugs 1 (4%) Antidepressants 2 (9%)	Antimotility drugs 8 (32%) Antispasmodic drugs 1 (4%) Antidepressants 3 (12%)
El-Salhy_a, 2019	17 years (9-25 years)		Yes	PPI medication 20 (36.4), Birth-control medication 29 (52.7), Antimigraine medication 5 (9.1), Medication against asthma/allergies 7 (12.7), Medication with levothyroxine 0 (0), Medication with heart/vascular drugs 2 (3.6)	PPI medication 21 (38.2), Birth-control medication 25 (45.5), Antimigraine medication 3 (5.5), Medication against asthma/allergies 6 (10.9), Medication with levothyroxine 1 (1.8), Medication with heart/vascular drugs 3 (5.5)	NR	NR
El-Salhy_b, 2019	17 years (9-25 years)		Yes	PPI medication 18 (32.7), Birth-control medication 30 (54.7), Antimigraine medication 4 (7.3), Medication against asthma/allergies 5 (9.1), Medication with levothyroxine 2 (3.6), Medication with heart/vascular drugs 1 (1.8)	PPI medication 21 (38.2), Birth-control medication 25 (45.5), Antimigraine medication 3 (5.5), Medication against asthma/allergies 6 (10.9), Medication with levothyroxine 1 (1.8), Medication with heart/vascular drugs 3 (5.5)	NR	NR
Halkjaer, 2018	NR	NR	No	Birth control pills 3 (12.0), PPI 2 (8.0), Antimigraine 1 (4.0), Asthma allergies 6 (24.0), Painkiller 4 (16.0), Circulatory 2 (8.0), Antiviral 1 (4.0), Topical steroid 1 (4.0), Laxatives 1 (4.0), Constipation drugs 1 (4.0), Other medications 1 (4.0)	Birth control pills 6 (23.1), PPI 6 (23.1), Antimigraine 1 (3.8), Asthma allergies 3 (11.5), Painkiller 4 (15.4), Antiviral 1 (3.8), Topical steroid 1 (3.8), Laxatives 1 (3.8), Constipation drugs 2 (7.7), Other medications 2 (7.7)	Continue use of IBS medication 14 (56.0) Former tried IBS medication 23 (92.0)	Continue use of IBS medication 15 (57.7) Former tried IBS medication 20 (76.9)
Holster, 2019	Unknown: n = 0, 1-5 year: n = 4, 5 years up: n = 4	Unknown: n = 1, 1-5 year: n = 3, 5 years up: n = 4	Yes	NR	NR	Laxatives 1, Antidiarrheal 1, Antispasmodic 1, SSRIs 5	Laxatives 2, Antidiarrheal 1, Antispasmodic 1, NaSSAs 2, SSNRIs 1, TCAs 1
Holvoet, 2021	10	7	Yes	NR	NR	Psychotropic drug use 8 (19%)	Psychotropic drug use 3 (16%)
Johnsen, 2017	10	10	Yes	NR	NR	Tricyclic antidepressant 1, loperamide 1	loperamide 1
Lahtinen, 2020	NR	NR	No	NR	NR	NR	NR

Supplementary Table 2: Summary of the descriptive characteristics of the included studies (Continued)

Author, year	Fecal microbiota transplantation						
	Fecal microbiota preparation	Placebo/ standard regimens	FMT route	Frequency and duration	Length of follow up	Adverse events, n (%)	
						FMT	Placebo
Aroniadis_a, 2019	75 FMT capsules containing 50 g feces from 1 of 4 donors	Placebo capsules (non-toxic brown pigment)	Water soluble gelatin oral capsules	25 capsules/day x 3 days	3 months	23 (47.9%)**	24 (50.0%)**
Aroniadis_b, 2019	75 FMT capsules containing 50 g feces from 1 of 4 donors	Placebo capsules (non-toxic brown pigment)	Water soluble gelatin oral capsules	25 capsules/day x 3 days	3 months	24 (50.0%)**	23 (47.9%)**
El-Salhy_a, 2019	30g of frozen donor stools with 40 ml isotonic saline	Own feces	Gastroscope: administer to distal duodenum	Once	3 months	48 (88.9%)	12 (21.7%)
El-Salhy_b, 2019	60g of frozen donor stools with 40 ml isotonic saline	Own feces	Gastroscope: administer to distal duodenum	Once	3 months	32(58.2%)	12 (21.7%)
Halkjaer, 2018	300 FMT capsules containing 144g fecal matter derived from 600g pooled donor feces	Placebo capsules (saline, glycerol, food coloring)	Capsules: Acid-resistant oral capsules	25 capsules/day x 12 days	6 months	22 (84.6%)	15 (57.7%)
Holster, 2019	30 g fresh donor feces with isotonic saline and 10%glycerol total 150ml). The fecal suspension was stored at -80 °C until use.	Own feces	Colonoscopy: administer to caecum	Once	6 months	4 (50.0%)	7 (87.5%)
Holvoet, 2021	Fresh donor feces with 300 mL isotonic saline mixed with a handheld blender and glycerol.	Own feces	NJ probe: administer to distal duodenum or proximal jejunum	Once	12 months	NR	NR
Johnsen, 2017	50-80 g fresh or frozen donor feces with 200ml isotonic saline and 50ml 85% glycerol	Own feces	Colonoscopy: administer to caecum	Once	12 months	3 (5.3%)	3 (10.0%)
Lahtinen, 2020	30g of stool donor with isotonic saline. The fecal suspensions from a single universal donor were prepared and stored at −80°C until the day of the treatment.	Own feces	Colonoscopy: administer to caecum	Once	12 months	7 (30.4%)	10 (40.0%)

*Abbreviations:NR, not reported

** *The data represent all patients during the period they were receiving FMT or placebo capsules over the entire course of the trial, before and after crossover
