# Supplementary material for: Fecal microbiota transplantation in irritable bowel syndrome: A meta-analysis of randomized controlled trials
Source: Front Med (Lausanne). 2022 Nov 3;9:1039284. doi: 10.3389/fmed.2022.1039284 (PMC9669599; doi:10.3389/fmed.2022.1039284)
Supplement: Supplementary file 11 [file Table_3.pdf]

Supplementary Table 3: inclusion and exclusion criteria of donors

Author, year	Number of donors	Inclusion criteria	Exclusion criteria
Aroniadis_a, 2019<br>Aroniadis_b, 2019	4	NR	NR
El-Salhy_a, 2019<br>El-Salhy_b, 2019	1	NR	- Use of antibiotics or other drugs that may impair gut microbiota, new sexual partners or travels abroad since the last screening<br>- Use of illegal drugs<br>- History of, or known exposure to, HIV, HBV, HCV, syphilis, human T-lymphotropic virus I and II, malaria, trypanosomiasis, or tuberculosis<br>- History of IBS, IBD, functional chronic constipation, coeliac disease, other chronic GI disorders, or chronic & systemic autoimmune disorders with GI involvement<br>- History of, or high risk for, GI cancer or polyposis<br>- History of neurological/neurodegenerative disorders, psychiatric conditions<br>- Known systemic infection not controlled at the time of donation<br>- Risky sexual behavior (anonymous sexual contacts; sexual contacts with prostitutes, drug addicts, individuals with HIV, viral hepatitis, syphilis; work as prostitute; history of sexually transmittable disease)<br>- Risk of transmission of diseases caused by prions<br>- Previous reception of tissue/organ transplant<br>- Previous (<12 months) reception of blood products<br>- Recent (<3 months) exposure to antibiotics, immunosuppressants, chemotherapy<br>- Recent (<6 months) needle stick accident, body tattoo, piercing, earring, acupuncture, travel in tropical countries, countries at high risk of communicable diseases or travelers' diarrhea, history of vaccination with a live attenuated virus, if there is a possible risk of transmission<br>- Recent parasitosis or infection from rotavirus, Giardia lamblia or other microbes with GI involvement<br>- Recent medical treatment in poorly hygienic conditions<br>- Recent appearance of diarrhea, hematochezia<br>- Recent ingestion of a substance that may result harmful for the recipients<br>- Healthcare workers (to exclude the risk of transmission of multidrug-resistant organisms)<br>- Individual working with animals (to exclude the risk of transmission of zoonotic infections)<br>- Overweight or obesity (body mass index > 25 kg/m^2)<br>- Chronic therapy with proton pump inhibitors<br>- Newly appeared GI signs or symptoms, for example, diarrhea, nausea, vomiting, abdominal pain, jaundice, illness or general signs as fever, throat pain, swollen lymph nodes<br>- Travel in tropical areas—contact with human blood (sting, wound, showing, piercings, tattoos)—sexual high-risk behavior<br>- Diarrhea (more than three loose or liquid stools per day) among members of the entourage (including children) within 4 weeks of donation<br>- Positive stool test for ova, parasites, *Salmonella* spp., *Shigella* spp., *Campylobacter* spp., *Yersinia* spp., toxin-producing *Clostridioides difficile*, *Aeromonas* spp., *Strongyloides*, or rotavirus<br>- Positive blood test for HIV, HAV, HBV, HCV, or *Treponema pallidum*
Halkjaer, 2018	4	- Age of 18-45 years<br>- Previous and currently healthy<br>- BMI 18.5-24.9<br>- Normal bowel movement (1-2 per day and type 3-4 at Bristol Stool Form scale)<br>- No medication consumption	- Antibiotic treatment in the past 6 months<br>- New tattoo or piecing within the last 6 months<br>- Born by Cesarean section<br>- Family history of GI diseases, cancer, diabetes, obesity, autoimmune diseases, allergy, asthma, eczema, cardiovascular diseases, neurologic or mental illnesses.<br>- Smoking, abuse of alcohol, or drugs<br>- Participation in high-risk sexual behavior, known or high risk of infectious diseases such as HIV, HAV, HBV, or HCV<br>- Allergy, asthma, or eczema<br>- Positive stool test for parasites or other enteropathogens, or *C. difficile* toxin
Holster, 2019	2	NR	- Antimicrobial treatment within last 6 months<br>- Tattoo or body piercing obtained within the 6 months before screening<br>- History of major gastrointestinal surgery<br>- Known organic gastrointestinal disease<br>- High-risk sexual behaviors, known or high risk of infectious diseases such as HIV or hepatitis<br>- Use of immunosuppressive or chemotherapy agents<br>- Abuse of alcohol or drugs<br>- Current communicable diseases<br>- Gastrointestinal polyposis, gastrointestinal malignancy, or non-gastrointestinal malignancy<br>- Eosinophilic disorders of the gastrointestinal tract<br>- Dementia, severe depression, or major psychiatric disorder<br>- Severe or morbid obesity, metabolic syndrome, autoimmune diseases, allergies, or chronic pain syndromes<br>- Pregnancy or breast-feeding<br>- Travelling to areas with endemic diarrhea during 3 months before screening<br>- Positive stool for ova, parasites, Giardia antigen, cryptosporidium antigen, *Clostridium difficile* toxin, *Salmonella*, *Shigella*, *E. coli* (EIEC, ETEC, EHEC), *Yersinia enterocolitica*, *Vibrio* or *Plesiomonas shigelloides.*<br>- Positive blood tests for HIV, Hepatitis A, B, or C
Holvoet, 2021	2	- Age of 18-65 years<br>- No gastrointestinal symptoms<br>- Normal bowel movement	- Antibiotic in the past 6 months<br>- BMI > 30 kg/m^2, chronic disease<br>- Detection of carbapenemase-producing enterobacterales or extended-spectrum beta-lactamase-producing orgamisms<br>- Positive blood tests for HIV, Hepatitis A, B, C, E or *Treponema pallidum*<br>- Positive stool test for parasites (eggs, cysts, or larvae), *Salmonella* spp., *Shigella* spp., *Campylobacter* spp., *Yersinia pseudotuberculosis*, C *difficile*, C *difficile* toxin, *Aeromonas* spp., or *Strongyloides*

Author, year	Number of donors	Inclusion criteria	Exclusion criteria
Johnsen, 2017	2	NR	- Antibiotic in the past 3 months<br>- New tattoo or piecing in the past 3 months<br>- High-risk sexual behavior<br>- Former imprisonment<br>- Chronic diarrhea, chronic constipation, or inflammatory bowel disease<br>- Colorectal polyps or cancer<br>- Immunosuppression<br>- Obesity, or metabolic syndrome<br>- Atopic skin disease<br>- Chronic fatigue<br>- Positive stool test for parasites, ova, cysts, *Salmonella* spp., *Shigella* spp., *Campylobacter* spp., *Yersinia* spp., toxin-producing C. *difficile*, *Helicobacter pylori* antigen, norovirus, rotavirus, Sapovirus, or adenovirus<br>- Positive blood test for HIV, Hepatitis A, B, C, or *Treponema pallidum*
Lahtinen, 2020	1	- No intestinal symptoms	- Antibiotic in the past 6 months<br>- Use of illicit drugs<br>- History of high-risk sexual behavior<br>- Recently travelled to areas with high incidence of infectious diarrhea.<br>- Positive blood tests for HIV, Hepatitis A, B, or C<br>- Positive stool culture for fecal bacterial pathogens (*Salmonella* spp., *Yersinia* spp., or *Campylobacter* spp.), or antibiotic-resistant bacteria (MRSA, ESBL)<br>- Positive stool test for *Clostridioides difficile* toxins, *Helicobacter pylori*, or parasite (ova or protozoa)

Abbreviation: NR, Not reported; GI, gastrointestinal; IBD, irritable bowel syndrome; IBD, inflammatory bowel disease; spp., species; ESBL, extended-spectrum beta-lactamases; MRSA, Methicillin-resistant Staphylococcus aureus;
