# Supplementary material for: Fecal microbiota transplantation in irritable bowel syndrome: A meta-analysis of randomized controlled trials
Source: Front Med (Lausanne). 2022 Nov 3;9:1039284. doi: 10.3389/fmed.2022.1039284 (PMC9669599; doi:10.3389/fmed.2022.1039284)
Supplement: Supplementary file 12 [file Table_4.pdf]

**Supplementary Table 4:** Comparison between donor and patient microbiome profile at pre FMT treatment and post FMT treatment

| Author, year | Microbiome measurement method | | | | Baseline microbiome profile between donor and patient | Microbiome profile after FMT treatment | FMT administration/type of feces | Global symptom of IBS |
|---|---|---|---|---|---|---|---|---|
| | α-Diversities | β- Diversities | Microbial richness | Dysbiosis Index | | | | |
| Aroniadis_a, 2019<br>Aroniadis_b, 2019 | Shannon diversity index | The Jensen-Shannon divergence | N/A | N/A | N/A | FMT recipients' microbiome diversity shift to the donors<br><br>Diversity among FMT responders was not different from non-responders. | FMT capsules / FMT capsules | Not improved |
| El-Salhy_a, 2019<br>El-Salhy_b, 2019 | N/A | N/A | N/A | Yes | Donor: dysbiosis index (DI) of 1, indicating normobiosis.<br><br>Patient microbiome profile's dysbiosis index prior to transplantation were 2.6, 2.8, and 2.7 in the placebo, 30g FMT, and 60g FMT groups, respectively | The DI values after transplantation were 2.6, 2.6, and 2.4, in the placebo, 30g FMT, and 60g FMT groups, respectively indicating that no significant change of DI among all three groups | Gastroscope/ Frozen feces | Improved |
| Halkjaer, 2018 | Chao1 index | Unweighted Unifrac | N/A | N/A | Fecal donors had a more diverse microbiome than patients with IBS | FMT recipients increase microbiome diversity shift to the donors at 12 weeks | FMT capsules/ FMT capsules | Not improved |
| Holster, 2019 | Shannon diversity and Pearson similarity index | N/A | N/A | N/A | donor microbiota diversity was not significantly different from patient microbiota diversity at baseline.<br>The abundance of butyrate-producing bacteria in the patient was not lower than in the donor group. | FMT recipients increase microbiome diversity shift to the donors ( but not significant)<br><br>No significant change in diversity of butyrate-producing bacteria | Colonoscope/ Fresh feces | Not improved |
| Holvoet, 2021 | Inverse Simpson index | N/A | N/A | N/A | Donor microbiome diversity was higher than patient microbiome diversity | N/A | Nasojejunal probe/ Fresh feces | Improved |
| Johnsen 2017 | N/A | N/A | N/A | N/A | N/A | N/A | Colonoscope/ Fresh and Frozen feces | Improved |
| Lahtinen, 2020 | N/A | N/A | Yes | N/A | Donor microbiome richness was higher than patient microbiome richness | Significant shift in the microbiota profile and richness | Colonoscope/ N/A | Not improved |

Abbreviations: N/A, No comparison microbial diversity between patient and donor; OTU, operational taxonomic unit; DI, dysbiosis index( DI-=1; normobiosis, DI >2; dysbioisis)
