# Supplementary material for: Fecal microbiota transplantation in irritable bowel syndrome: A meta-analysis of randomized controlled trials
Source: Front Med (Lausanne). 2022 Nov 3;9:1039284. doi: 10.3389/fmed.2022.1039284 (PMC9669599; doi:10.3389/fmed.2022.1039284)
Supplement: Supplementary file 13 [file Table_5.pdf]

frontiers

Supplementary Table 5: The association of gut microbiota profiles and global symptom improvement

Author, year	Global outcome of IBS	Donor microbiome profile	Patient microbiome profile baseline	Patient microbiome profile after FMT
Aroniadis_a, 2019	Not improved	N/A	- A baseline high abundance (>5%) of Prevotella was detected in seven patients, six of whom responded to treatment<br>-The highest relative abundance of Bacteroidetes to Firmicutes was observed in FMT responders at baseline	N/A
Aroniadis_b, 2019				
El-Salhy_a, 2019	Improved	- high level of microbial diversity (normobiotic) with favorable bacteria signature ((Streptococcus, Dorea, Lactobacillus and Ruminococcaceae spp.)	N/A	IBS severity scoring system (IBS-SSS) was shown to be strongly associated with signals from the Lactobacillus and Alistipes species
El-Salhy_b, 2019				
Halkjaer, 2018	Not improved	microbial diversity of donor was not related to global symptoms.	N/A	Blautia genus of the Clostridiales correlating negatively with IBS-SSS .<br>The Bacteroides genus and Ruminococcaceae family was positive correlations with IBS-SSS.
Holster, 2019	Not improved	N/A	N/A	Patient with a positive symptom response did not show increased fecal or mucosal microbiota diversity.
Holvoet, 2021	Improved	N/A	Patients who responded to active FMT had a greater baseline microbial diversity than those who did not.	N/A
Johnsen, 2017	Improved	N/A	N/A	N/A
Lahtinen, 2020	Not improved	N/A	N/A	N/A

Abbreviations: √, improve global outcome of IBS; x, not improve global outcome of IBS; N/A, No comparison microbial diversity between microbiome profile and global symptom improvement; OTU, operational taxonomic unit
